# Biological Responses to Combined Nanoparticles: Uptake, Distribution and Toxicity

**DOI:** 10.3390/nano16110695

**Published:** 2026-06-02

**Authors:** Lu-Lu Chen, Jun-Hao Guo, Yuan-Yuan Liu, Haifang Wang

**Affiliations:** Institute of Nanochemistry and Nanobiology, Shanghai University, Shanghai 200444, China; clulu@shu.edu.cn (L.-L.C.); guojunhao810@shu.edu.cn (J.-H.G.)

**Keywords:** co-exposure, nanomaterials, cellular uptake, mixed toxicity, biodistribution

## Abstract

The biological effects of nanoparticles (NPs) form the basis of their safety assessments and biomedical applications. However, most related studies have focused on exposing biological systems such as cells and animals to individual NPs. This is far removed from real-world environmental exposure and biomedical application scenarios involving NPs. In practice, NPs often coexist with other types of NPs or the same type of NPs of different sizes. Interactions between mixed NPs can alter their dispersion states and biological behaviors, thereby influencing their cellular internalization, distribution, and ultimately determining their toxicity outcomes. In this review, we summarize the research progress and current understanding of the biological effects of mixed NPs. We focus on how co-exposure influences the uptake/absorption, fate, and toxicity of NPs in cells and animals. Co-exposure results in an increased, decreased, or unaffected cellular uptake of NPs by altering their dispersion states and protein corona in biological media, and thus their uptake routes. Cytotoxicity of mixed NPs exhibits patterns of synergistic, antagonistic, or additive effects, and is not always positively correlated with the intracellular contents of the NPs, highlighting the complexity of the response of biological systems to NP co-exposure. In vivo evidence further indicates that co-exposure to NPs can result in alterations in the absorption efficiency, tissue distribution, and clearance of the NPs, and thus their overall toxicity. Finally, we discuss the limitations of the current research on the biological response to mixed NPs, and propose key challenges and future directions towards a more standardized, mechanism-based assessment of NP mixtures.

## 1. Introduction

The rapid development of nanotechnology has led to the massive production of a variety of nanomaterials. These nanomaterials are now extensively applied across diverse sectors, including industrial manufacturing [1], biomedicine [2], environmental remediation [3], and various other areas of human production and life [4,5,6,7]. According to StatNano (https://product.statnano.com), as of 31 March 2026, the Nanotechnology Products Database had monitored 193 nanomaterials used in over 10,000 products produced in 68 countries. Driven by widespread application, production volumes have been increasing over years. For example, 2025’s projected graphene production is 14 times higher than 2015’s, whereas the expected 2030 production (3461 tons) is 24 times higher than 2020’s [8]; Globally, the annual production of silver (Ag) nanomaterials was 55 tons around 2012 and increased to 400 tons around 2023 [9,10]. Beyond large-scale production, environmental accumulation is equally concerning. Using a stochastic dynamic model NanoRelease, Song et al. predicted that the annual releases of titanium dioxide (TiO_2_), silica and iron oxide (FeOx) nanomaterials in 2020 reached over 10,000, 4000 and 5000 tons, respectively [11]. As nanomaterials are continually produced, applied and deposited into the environment, humans are increasingly exposed to them, either intentionally or unintentionally. This has raised concerns about their safety.

Extensive research on biological responses to nanoparticles (NPs) has shown that they affect microorganisms, plants and animals in different ways at molecular, cellular, tissue, and whole organism levels [12]. Furthermore, NPs show adverse effects on various aspects of human health, including the blood system, nervous system and reproductive system [13]. Through in vitro experiments using human platelets and mice, Singh et al. demonstrated that graphene oxide induced platelet aggregation and led to pulmonary thromboembolism [14]. Similarly, Liu et al. observed that silica NPs (SNPs) could disrupt blood–brain barrier structure and induce barrier inflammation in vitro and in vivo [15], and Khodadadi et al. reported that TiO_2_ NPs reduced testosterone levels and sperm quality while causing testicular damage in mice [16].

However, in real-world environments, NPs often coexist with various other contaminants rather than existing in isolation. The potential interactions between NPs and coexisting contaminants may alter the bioaccumulation and/or toxicity of the NPs, the coexisting contaminants, or both. Many studies have investigated the toxicity of NPs in the presence of various contaminants, such as heavy metals [17,18,19] and organic compounds [20,21,22,23]. Beyond traditional pollutants, the ubiquitous use of nanomaterials means that different types of NPs now frequently coexist with one another at varying exposure levels. As a result, complex mixtures of diverse NPs are routinely detected in municipal wastewater treatment systems [7,8,9,24] and, subsequently, in receiving waters and soils [25,26,27,28,29,30]. In addition, NPs are not of a single size, but typically exhibit a certain range of sizes [31]. Because size fundamentally dictates physicochemical properties, differently sized NPs of the same composition can elicit distinct biological responses [32,33], effectively acting as multiple, separate contaminants. Consequently, organisms are exposed to different types and sizes of NPs simultaneously.

Do the biological effects under mixed NP exposures differ from those of individual NPs? Are there any new biological effects for individual NPs under co-exposure? Although related research is relatively limited, it has been found that exposure to mixed NPs probably results in alterations to the biological response of the NPs. A review of the toxicity of NP mixtures to various aquatic organisms and plants shows that of cases displaying joint toxic responses, 53% showed antagonistic effects, 25% showed synergistic effects, and 22% showed additive effects [34]. These environmental studies highlight the complexity of combined biological responses. Therefore, understanding the combined impact of multiple NPs on humans is necessary for assessing the human health risk posed by nanomaterials [35]. This is also important for nanomedicine formulations in therapeutic contexts.

In this review, we summarize the progress of the research on the effect of co-exposure to mixed NPs on eukaryotic cells and animals, focusing on the uptake, distribution and toxicity of mixed NPs. 

## 2. Methodology of Search Strategy

Data were primarily gathered from peer-reviewed literature indexed in the Web of Science database. A systematic search was performed using a broad set of keywords combining exposure terms (“co-exposure”, “combined exposure”, “combined toxicity”, “joint exposure”, “*mixture”, “simultaneous exposure”, “mixed exposure”, “binary mixture”) with “nano*”, “graphene”, or “quantum dot”. No restriction on publication date was applied to ensure comprehensive coverage. In addition to the database search, citation tracking of relevant review articles and original research papers was performed to identify further eligible studies. To ensure the timeliness of the literature base, an updated search was conducted prior to submission.

Based on these search terms, we obtained candidate publications and manually screened the results to retain studies that met the following inclusion criteria: (1) The study reported both single-exposure and co-exposure data, thereby enabling direct comparison. (2) The study provided NP characterization data, at minimum including electron microscopy size and hydrodynamic size/suspension stability; the electron microscopic size of at least one type of NP is ≤100 nm. (3) The study was conducted on mammalian cells or mammalian animal models. Studies focusing exclusively on bacteria, plants, algae, aquatic organisms, or other non-mammalian species were excluded.

Totally, 44 papers are included in this review, including 27 cell experiments, 15 animal studies, and 2 studies involving both cell and animal experiments. We note that the available literature is unevenly distributed across NP types, with a predominance of studies on polystyrene (PS), Ag, silica, zinc oxide (ZnO), FeOx, and TiO_2_ NPs. However, studies on some important nanomaterials, e.g., 2D materials, hybrid nanostructures and clinically relevant nanomedicines, remain scarce. This reflects the current research landscape and calls for deeper exploration in the future.

## 3. In Vitro Responses to Mixed NPs

As with single NP exposure, cell models are the preferred choice for researchers seeking to reveal the cellular uptake and toxicity of mixed NPs. This is because a cell model is a simplified representation of the vastly more complex in vivo environment, which is a necessary step towards modeling real-life exposure. Furthermore, the results obtained for mixed NPs can be compared with those obtained for individual NPs. As the accumulation of NPs in cells can cause adverse effects and affect cell function, leading to toxicity in the human body [36,37], it is crucial to understand the cellular uptake of mixed NPs. Lamoree et al. emphasized the importance of confirming the presence or uptake of particles in the cells or organs in studies investigating the potential harmful effects of NPs [38].

Similarly to the effects of mixed NPs on aquatic organisms and plants [34], various combined responses have been observed in eukaryotic cells, including antagonistic, synergistic, and additive effects, although synergistic effects were dominant. In addition, while the cellular uptake of NPs is generally positively proportional to their toxicity, in some cases altered toxicity of NPs is not associated with change in their cellular content. Different mechanisms have been proposed to explain the altered cellular uptake and toxicity of different NP mixtures.

Under co-exposure, NPs can adopt markedly different physicochemical states—well dispersed, homoaggregated, heteroaggregated, or coated with distinct biomolecular coronae—depending on the interplay of three interconnected physicochemical factors. First, electrostatic interactions arise when NPs with opposite or significantly different surface charges undergo electrostatic attraction to form larger assemblies with reduced colloidal stability, while similarly charged NPs may compete for charged membrane binding domains. Second, hydrophobic interactions influence the dispersion state of NPs and the composition of the protein corona on NPs in biological fluids, thereby affecting NP-cell recognition. Third, surface functionalization, including PEGylation, carboxylation, or peptide conjugation, can inhibit or promote inter-NP interactions and alter the engagement of specific endocytic pathways. These factors collectively determine whether NPs remain dispersed or form aggregates, including heteroaggregation between dissimilar NPs, which can alter their effective size, morphology, and membrane interactions.

In parallel, the biomolecular corona that rapidly forms on NP surfaces introduces an additional layer of regulation: NPs compete for available biomolecules such as serum proteins and cell-secreted biomolecules, leading to distinct biological identities that modulate their affinity for specific membrane receptors, and the corona undergoes dynamic exchange, known as the Vroman effect, which may further alter NP-cell recognition over time [39,40,41]. Together, these aggregation-driven and corona-mediated processes collectively orchestrate how mixed NPs engage with cellular entry machinery.

These complex, interrelated mechanisms give rise to diverse patterns of cellular uptake and toxicity under co-exposure, as detailed in the following sections.

### 3.1. Cellular Uptake

Cellular uptake of NPs is the process by which cells transport extracellular NPs across the plasma membrane into the cell. This process is governed by dynamic interactions between NPs and the cell membrane, enabling NPs to enter the cell through multiple pathways in an energy-independent and/or dependent manner. Most NPs enter cells via energy-dependent pathways, which are broadly classified into phagocytosis and pinocytosis. Pinocytosis can be further subcategorized into clathrin-mediated endocytosis (CME), caveolin-mediated endocytosis, clathrin-/caveolae-independent endocytosis and macropinocytosis [42]. NP uptake is generally time- and concentration-dependent and greatly affected by their basic physicochemical properties, including size, shape, charge, colloidal stability, and the ability to adsorb environmental compounds (such as proteins) [35,43]. The interplay between these physicochemical factors determines whether the mixed NPs still remain in their dispersion state or aggregate/disaggregate. Particularly, when NPs form large aggregates (e.g., >500 nm) under co-exposure conditions, their cellular internalization may no longer follow classical nanoscale endocytic pathways (such as CME) but rather proceed via phagocytosis or passive sedimentation-driven cell association. Distinguishing endocytosis from passive deposition and aggregate engulfment is critical for mechanistic interpretation, yet this distinction is frequently missing in the literature.

**Figure 1:**
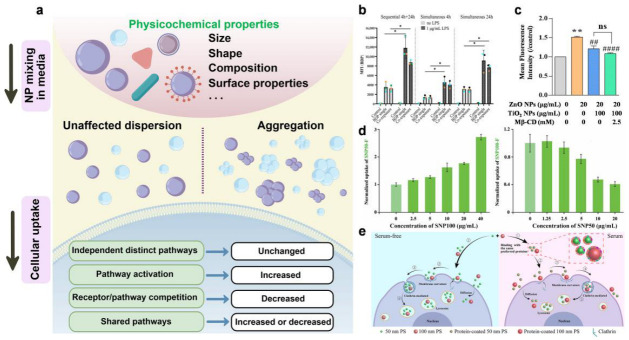
Uptake of NP mixtures by cells under co-exposure. (**a**) Schematic summary of the major mechanisms regulating NP uptake under co-exposure. (**b**) Co-exposure to 920 nm silica particles did not result in alterations in the uptake of 59 nm SNPs by macrophages, even under LPS-stimulated inflammatory conditions (* *p* < 0.05 compared to the control group) [56] (with permission of MDPI). (**c**) TiO_2_ NPs reduced ZnO NP uptake in HaCaT cells under co-exposure (** *p* < 0.01 compared to the control group. ^##^ *p* < 0.01, ^####^ *p* < 0.0001 compared to the single ZnO NP group) [46] (with permission of MDPI). (**d**) SNP100 promoted the uptake of SNP50, whereas SNP50 showed weaker or inhibitory effects on SNP100 [45] (with permission of Wiley-VCH Verlag). (**e**) Schematic illustration of the uptake of mixed-sized PS NPs under serum-free and serum-containing conditions [39] (with permission of Elsevier).

Consequently, exposure to mixed NPs can lead to different effects on NP uptake by cells, including increased [44,45], decreased [46,47] or unaffected [48,49] uptake compared to single exposures (Table 1). This highlights the complexity and uncertainty of cellular uptake behavior in co-exposure scenarios. Compared to single exposure, the endocytic pathways engaged under co-exposure can undergo substantial changes, as described in the four scenarios outlined below (Figure 1a). Physical blocking of cell membranes also hinders NPs from reaching cellular entry sites and thus reduces their cellular uptake [50]. In addition, proteins secreted by cells can form protein coronas on the surface of NPs, which alter their dispersion stability and adhesion ability to cell surfaces, ultimately changing their cell uptake efficiency [40]. These mechanisms together lead to distinct differences in NP uptake behaviors under single and co-exposure conditions.

#### 3.1.1. Unaffected NP Uptake Through Independent Pathways

When different types of NPs enter cells via independent, non-overlapping endocytic pathways during co-exposure, their cellular uptake levels are comparable to those during single exposure. Cells internalize each type of NP independently, without competing for endocytosis-related components. For example, Tsugita et al. found that co-exposure to SNPs and TiO_2_ NPs (both less than 50 nm) did not result in a significant change in their uptake by mouse bone marrow-derived macrophages (BMDMs) compared to single exposure [58]. Furthermore, Susnik et al. utilized confocal laser scanning microscopy to observe the uptake of colloidally stable 59 nm SNPs and 920 nm silica particles in both unstimulated and LPS pre-stimulated macrophages (J774A.1) and no significant differences were observed between single and co-exposure conditions after 24 h of incubation (Figure 1b) [56]. In line with these observations, Ilić et al. reported that ~67 nm Ag NPs and ~17 nm PS NPs were internalized independently by human Jurkat T lymphocytes without mutual interference. Confocal imaging confirmed no colocalization between Ag NPs and PS NPs in the cytoplasm [60], supporting separate endocytic pathways, and the uptake efficiency of either NP was not altered in the presence of the other.

#### 3.1.2. Increased NP Uptake by Activating Additional Pathways

Some types of NPs can actively trigger specific endocytic pathways (e.g., macropinocytosis or crosstalk between compensatory pathways), thereby enhancing the internalization of the other type of NPs that would otherwise exhibit low uptake efficiency. Vanhecke et al. compared the uptake of polymer-coated 4.7 nm Au NPs and 13.6 nm iron oxide NPs in mouse macrophages (J774A.1) under single and co-exposure for 24 h [57]. While observing continuous uptake of both NP types under all exposure conditions, they observed that the 24 h uptake increased by ~31% for Au NPs and ~37% for iron oxide NPs under co-exposure relative to single exposure. This was attributed to faster internalization of the NPs under co-exposure relative to single-exposure experiments. They found that the uptake of the NPs involved CME and at least one additional endocytic pathway. However, co-exposure to the two types of NPs in the presence of the CME inhibitor failed to suppress their uptake. This overcoming of the expected inhibition suggested a compensatory crosstalk between CME and an alternative endocytic pathway [57]. The authors concluded that co-exposure resulted in an increase in the uptake regardless of NP type.

Activation of certain endocytic pathways can be achieved by functionalizing NPs with specific groups (such as the cell-penetrating peptide TAT, the transactivator of transcription). TAT can trigger the receptor (e.g., HSPG)-mediated macropinocytotic pathway. Wei et al. reported that TAT-NPs can trigger the macropinocytotic pathway, enabling the other type of “bystander” NPs to be co-ingested by cells [53]. Ag NPs (~50 nm) alone showed very low cellular uptake by Chinese hamster ovary cells; however, the addition of TAT-Ag NPs increased the uptake of bystander Ag NPs to approximately 10~80 times that of the Ag NPs alone. This is a unique indirect mechanism by which NPs can influence the uptake of surrounding NPs. NPs including Ag, Au, iron oxide and quantum dots have been functionalized with TAT to enhance the uptake of other NPs that have difficulty in entering cells alone, such as bare Ag NPs, Au NPs, iron oxide NPs, and quantum dots of different sizes in various cell lines [53]. Although this effect is influenced by NP composition and concentration, as well as cell type, approximately 50 nm bystander NPs consistently demonstrate the highest uptake efficiency [54]. Using coarse-grained molecular dynamics simulations and theoretical energy analyses, the authors proposed a “membrane-curvature-mediated co-endocytosis” mechanism to explain the enhanced uptake of bystander NPs by TAT-NPs [63].

#### 3.1.3. Decreased NP Uptake Due to Receptors/Pathway Competition

When both types of NPs depend on the same receptors and/or endocytic pathways, the NPs with a stronger affinity will preferentially occupy the receptor-binding sites. This competitive inhibition effectively blocks the other NPs from binding and entering the cell, resulting in a decrease in their uptake. This universal mechanism functions across a wide range of NP types including TiO_2_, ZnO, Ag and PS NPs, as well as various cell lines. Its effect is often exacerbated by the enlargement of NP size induced by aggregation, which further compromises overall uptake efficiency. The final net uptake outcome depends sensitively on their affinities to binding sites, concentration and ratios, and the specific endocytic pathways involved (such as CME versus caveolin-mediated endocytosis). Liang et al. found that 30 nm TiO_2_ NPs (100 mg/L) significantly restricted the cellular uptake of 30 nm ZnO NPs (20 mg/L) in the human keratinocyte cell line (HaCaT) for 6 h. This inhibitory effect persisted regardless of whether the ZnO NPs were dispersed or aggregated, by competing for shared endocytic pathways, particularly caveolae-mediated endocytosis, and by promoting particle aggregation (Figure 1c) [46]. Notably, NP aggregation from pristine nanoscale sizes to larger micron-scale agglomerates has been widely documented to impair both transmembrane penetration and cellular uptake, amplifying the competitive inhibition effect in mixed NP systems. Similarly, mutual uptake inhibition has been observed between high-density polyethylene NPs and citrate-stabilized Ag NPs in human intestinal Caco-2 and HT29MTX cells, owing to their overlapping cellular entry mechanisms [55]. It should be noted that the authors examined the endotoxin contamination of NPs, and mentioned that the contamination of polyethylene NPs with endotoxins may affect cellular responses [55], as endotoxins can provoke inflammatory reactions and alter the endocytic pathways [56].

#### 3.1.4. Increased or Decreased NP Uptake by Sharing Pathways

When NPs of different sizes and/or compositions share a common endocytic pathway, their simultaneous engagement can result in either increased or decreased uptake depending on their size, concentration and ratio, as well as colloidal interactions. The net outcome reflects a balance between cooperative entry and competition for limited resources used to build the pathway (e.g., key proteins of membrane curvature-sensing proteins, clathrin, and receptors for CME).

In 2019, our group discovered a novel size-dependent effect on the cellular uptake of differently sized NPs under co-exposure: 100 nm SNPs significantly promoted the uptake of 50 nm SNPs by human cervical cancer cells (HeLa) in serum-free medium, while 50 nm SNPs slightly decreased the uptake of 100 nm SNPs (Figure 1d) [45]. Mechanistic investigations revealed that both 50 nm and 100 nm SNPs rely on CME as their shared entry pathway. Statistical analysis of thousands of cellular uptake events using transmission electron microscopy (TEM) and flow cytometry measurements revealed that a single 100 nm SNP particle can effectively trigger CME and carry nearby 50 nm SNP particles into the cell via CME, thereby enhancing the cellular uptake of 50 nm SNPs [44,45,51]. In contrast, although 50 nm SNPs enter cells through CME, about six 50 nm SNP particles are required to trigger a CME event. Therefore, 50 nm SNPs may compete with 100 nm SNPs for limited CME-associated receptor-binding sites or downstream endocytic components, resulting in a slight reduction in the uptake of 100 nm SNPs [44]. This co-endocytosis via a shared CME pathway results in an enhanced uptake of the smaller NPs but a decreased uptake of larger NPs.

de Boer et al. also observed this size-dependent enhancement, reporting increased uptake of 40 nm carboxylated PS NPs in HeLa cells over 24 h in a serum-containing medium, but unaffected or decreased uptake of 100 nm PS NPs depending on the PS concentrations [41]. More recently, we performed a systematic study on plain PS NPs of 50 nm (2.5–10 μg/mL) and 100 nm (10–60 μg/mL) at various concentrations and ratios in both serum-free and serum-containing media [39]. The cellular uptake of 50 nm PS NPs was generally enhanced by co-exposure with 100 nm PS NPs in both media types. This is consistent with the CME-triggering mechanism observed for SNPs, whereby one 100 nm PS NP can activate CME and facilitate the entry of nearby smaller PS NPs into cells. However, the uptake of 100 nm PS NPs was more complex, particularly in serum-containing medium. Both increase and decrease were observed depending on the concentration ratio and serum content. This bidirectional outcome reflects competition between the two types of NPs for the limited CME-initiating membrane proteins and preferred serum proteins that form distinct coronae on NPs, modulating their interactions with cell surface receptors (Figure 1e) [39]. Notably, this threshold-dependent CME triggered by NPs has been validated across different sizes of SNPs and PS NPs, as well as multiple cell lines, suggesting that it represents a general principle governing the cellular uptake of mixed-sized NPs [39,41,44,45,51]. The key variables that modulate this pattern are the presence of serum, which alters the composition of the protein corona of the NPs and thus their interactions with the cell membrane; the concentrations of each type of NP, and the ratio of the two types of NP in the mixture [39,41].

Using LC-MS/MS-based proteomic profiling of the protein corona formed on these PS NPs in serum-containing medium, we found that 50 nm and 100 nm PS NPs competed for binding to the same preferred serum proteins, such as protein C and transferrin. When the concentrations of the preferred proteins were insufficient to cover the total NP surface area, the competitive redistribution of these proteins between the two NP populations altered the corona composition and consequently modulated the affinity of 100 nm PS NPs for cell membrane receptors and their subsequent endocytosis [39]. These findings demonstrate that protein corona reshaping is a key mechanistic driver of altered cellular uptake under co-exposure, not merely a confounding factor. This reminds us that the formation of a biomolecular corona is a dynamic process [64]. Once NPs enter a biological medium, proteins and other biomolecules of varying abundance, affinity, and residence time are adsorbed and dissociated from the NP surface in a dynamic manner, forming soft and hard coronas. The soft corona is loosely associated and can rapidly exchange with surrounding biomolecules, whereas the hard corona is more strongly bound and may persist for longer periods, directly influencing cell recognition [65,66]. In reality, these are highly dynamic, continuously evolving interfacial processes. In NP mixtures, competition between different particle populations for the same biomolecular pool, different adsorption kinetics on different particle surfaces, and changes in aggregation or sedimentation that alter accessible surface area may further complicate these dynamic processes. Therefore, elucidating dynamic corona-mediated uptake under co-exposure conditions is an important area for future mechanistic studies.

In summary, co-exposure complicates matters with regard to the endocytic pathways of NPs, as these can be influenced by a variety of factors. The dynamic interactions between NPs can trigger the reconstruction of the endocytic regulatory network, leading to changes in cellular uptake behavior. The unpredictability of these dynamic interactions underscores the critical challenge and necessity of transitioning from traditional single-particle models to complex, multi-component exposure scenarios when evaluating the true biological effects of nanoparticles. The available evidence suggests that increased uptake is most likely when one population of NPs can efficiently trigger an endocytic pathway and promote the co-internalization of nearby NPs. This is also likely when moderate aggregation or protein adsorption enhances membrane contact without preventing internalization, or when pathway crosstalk activates alternative uptake routes. In contrast, decreased uptake is more likely to occur when NPs compete for the same membrane receptors or endocytic machinery, when key membrane-associated proteins become limiting, when agglomeration produces particles that are too large for efficient internalization, or when corona formation reduces membrane recognition. Therefore, the outcome of the uptake of NP mixtures should be interpreted as the balance between uptake-promoting and uptake-limiting factors rather than as a fixed property of a given NP pair.

It should be noted that there is substantial variability in characterization practices across studies. Since the composition of the medium, ionic strength, pH, serum proteins, natural organic matter, and coexisting particles can significantly affect the aggregation, sedimentation, dissolution, ion release, and corona formation of mixed NPs, characterization in water or stock suspensions alone may not accurately reflect the actual exposure state of NP mixtures. Future co-exposure studies should characterize NPs under actual exposure conditions and, where possible, over the exposure time course [67]. Key information should include hydrodynamic size, polydispersity index, zeta potential, sedimentation behavior, dissolution or ion release, delivered-to-cell dose, and corona characteristics. This information is essential for distinguishing true biological mechanisms from artifacts caused by poor colloidal stability or inconsistent dosimetry.

It should also be noted that the mechanistic assignment of NP uptake pathways is often methodologically challenging. Most studies rely on pharmacological inhibitors or colocalization analysis, but these approaches have intrinsic limitations [68]. For example, endocytic inhibitors may exhibit off-target effects, concentration-dependent cytotoxicity, incomplete pathway specificity, or indirect effects on membrane organization. Similarly, colocalization between NPs and pathway markers or intracellular compartments suggests possible trafficking routes, but does not prove a specific uptake mechanism in itself. Therefore, uptake mechanisms inferred from inhibitor or colocalization studies should be interpreted with caution and, where possible, validated using complementary approaches such as genetic knockdown/knockout and proteomic analysis.

### 3.2. Cytotoxicity

The cytotoxicity induced by NPs manifests at two primary levels. The first involves early alterations in functional and immunological parameters that have not yet directly manifested as cell death or apoptosis. These changes include cytokine/chemokine release (such as IL-1β, IL-6 and TNF-α), alterations in phagocytic/chemotactic function, activation of inflammatory signaling pathways (e.g., inflammasome/caspase-1, NF-κB and MAPK), overproduction of oxidative stress, and alterations in cell barrier function [69]. These changes are usually detected within hours to days of exposure and often appear before or independently of the endpoint of cell death, reflecting the response to “stress” or “danger signals”. Therefore, these changes are often regarded as early warning indicators of nanotoxicity. In co-exposure studies, changes in immune signals can be observed at lower doses [70,71]. When cumulative cellular damage exceeds the cell’s repair capacity, cell viability decreases, or programmed cell death may ensue. In other words, the second level refers to the changes in cell viability and death outcomes, such as apoptosis and necrosis.

Studies have shown that exposure to mixed NPs may lead to synergistic, antagonistic or unaffected cytotoxicity [34]. This variability in toxicity stems not only from the physicochemical properties of the NPs, but also from exposure dose/time, cell type, culture environment (e.g., serum/protein presence), and experimental endpoint (for example, differences in sensitivity between measurements of reactive oxygen species (ROS)/inflammatory markers and direct assessment of cell death). A co-exposure toxicity study on 12 different types of cells demonstrated that the mixture of solid lipid NPs (SLNs) and superparamagnetic iron oxide NPs (SPIONs) exhibited different toxic effects, ranging from synergistic and additive effects to antagonistic effect, in different cells, which was closely associated with mitochondrial dysfunction and oxidative stress [72]. Clearly, the toxic effects of NP mixtures cannot be reliably predicted from single exposures. Table 2 is a summary of cytotoxicity outcomes of mixed NPs. As the additive effect of mixed NPs means that the addition of the other type of NPs does not induce appreciable changes in the behavior of the NPs, i.e., the observed mixture response is close to the predicted combined effect [61,73,74], this section focuses primarily on synergistic and antagonistic cytotoxicity induced by NP mixtures and the underlying mechanisms.

#### 3.2.1. Synergistic Toxic Effect

**Figure 2:**
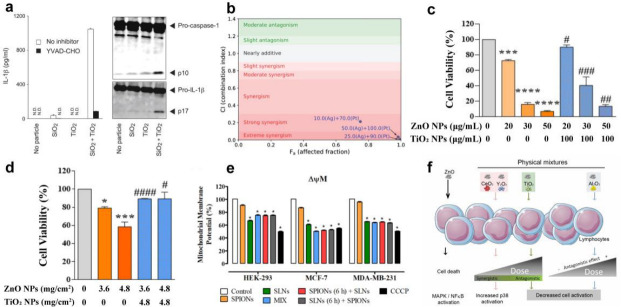
Cytotoxic effects of mixed NPs. (**a**) Co-exposure to SiO_2_ and TiO_2_ NPs results in IL-1β release and caspase-1/IL-1β cleavage in LPS-primed BMDMs [58] (with permission of BMC). (**b**) Combination index analysis of Ag/Pt NP co-exposure in hCMEC/D3 cells after 24 h treatment shows a strong synergism [48] (with permission of Wiley-VCH Verlag). (**c**) TiO_2_ NPs attenuated the cell viability loss of HaCaT cells induced by ZnO NPs (*** *p* < 0.001, **** *p* < 0.0001 compared to the control group, ^#^ *p* < 0.05, ^##^ *p* < 0.01, ^###^ *p* < 0.001 compared to the single ZnO NP group) [46] (with permission of MDPI). (**d**) TiO_2_ NPs antagonized the cytotoxicity caused by ZnO NPs in reconstructed human epidermis (EpiSkin) (* *p* < 0.05, *** *p* < 0.001 compared to the control group. ^#^ *p* < 0.05, ^####^ *p* < 0.0001 compared to the single ZnO NP group) [46] (with permission of MDPI). (**e**) Mixed SPIONs and SLNs induced different changes in mitochondrial membrane potential in human embryonic kidney cells (HEK-293), human breast adenocarcinoma cells (MCF-7), and human triple-negative breast cancer cells (MDA-MB-231) under different treatment conditions (* *p* < 0.05 compared to the control group) [72] (with permission of Sage Publications). (**f**) Schematic illustration of the various cytotoxic responses and signaling changes induced by mixed ZnO and other metal oxide NPs [79] (with permission of MDPI).

Synergistic toxicity refers to the situation where the combined toxicity of a mixture is significantly greater than the sum of the individual toxicities of each component. It has been widely reported in co-exposure studies involving NPs. Co-exposure to NPs may result in synergistic toxicity by triggering various biological responses, from exacerbated oxidative stress, enhanced immunomodulatory responses [48,58], enhanced degradation of the extracellular matrix [48], loss of cell stiffness [60], mitochondrial dysfunction [49], genotoxicity [61], to inhibition of proliferation [48], apoptosis and mortality [49]. At concentrations where individual SNPs and TiO_2_ NPs do not cause any toxicity in macrophages, their mixture activated caspase-1 inflammasome, inducing the production of the inflammatory factor IL-1β (Figure 2a) [58]. Particle-induced lysosomal stress and/or ROS are considered likely mechanisms for inflammasome activation. Although TiO_2_ NPs alone did not induce IL-1β, TiO_2_-induced ROS production may enhance SiO_2_-induced cellular stress leading to inflammasome activation. Notably, this synergistically induced inflammation was also observed in vivo [58]. Besides co-exposure to mixed NPs of different compositions, co-exposure to mixed NPs of the same composition but different sizes may also result in synergistic toxicity. As shown by the study conducted by Yang et al. [75], co-exposure to 800 nm and 100 nm PS particles resulted in more severe toxicity in human placental choriocarcinoma cell line JEG-3. Stronger oxidative stress and injury phenotypes, including higher ROS, lower CLDN3 (tight-junction related) level, higher percentage of cells at S phase, lower proliferation, and higher apoptosis were observed under co-exposure than under single exposures. Since mixed NPs of different sizes are commonly found in the environment, this highlights the importance of investigating the combined toxic effects of NPs with a range of sizes in the future.

Generally, synergistic toxicity induced by co-exposure to NPs is accompanied by enhanced uptake of the NPs and/or their dissolved species. Domenech et al. reported that the addition of PS NPs to low concentrations of Ag NPs would increase Ag accumulation and its genotoxicity and oxidative DNA damage [61]. Li et al. found that a non-toxic concentration (6.25 μg/mL) of ZnO NPs enhanced the toxicity of Cu NPs in Hep-G2 cells, and the enhanced cytotoxicity was attributed to the intracellular ZnO NPs, but not zinc ions [59]. We demonstrated that, compared with single exposure, co-exposure to 35 nm Ag NPs and 40 nm or 120 nm SNPs at individual non-toxic concentrations resulted in more severe cytotoxicity, as indicated by the overgeneration of ROS, decreased mitochondrial membrane potential, ATP depletion, and increased apoptosis/necrosis [52]. Combined effect evaluation on cell viability was characterized by the concentration addition index and the values were around 0.5–0.6 (>0) for the co-exposure to Ag NPs (10 μg/mL) and SNPs (5 μg/mL), indicating that the combined effects are synergistic. Dissolved Ag ions that are delivered into cells by adsorbing onto SNPs and directly internalized Ag NPs are the primary contributors to the combined toxicity.

In the meantime, some studies reported that synergistic toxicity is not driven by increased NP uptake. Korzeniowska et al. found that mixed Ag NPs and platinum NPs led to a strong synergistic inhibition of proliferation in human brain microvascular endothelial cells (hCMEC/D3) by triggering enhanced immunomodulatory responses and the degradation of the extracellular matrix [48]. The strong synergistic effect (Figure 2b) was obtained by quantitative evaluation using the Chou–Talalay combination index method, a classical combination-effect analysis framework adapted from drug-mixture studies, in which cell viability data were converted into affected fractions and plotted against combination index values to distinguish synergistic, additive and antagonistic interactions. At the same time, they found that co-exposure conditions did not result in enhanced uptake of either NP type. Similarly, Ilić et al. found that mixed Ag NPs and PS NPs exerted synergistic toxicity in human Jurkat T cells, as evidenced by exacerbated oxidative stress, apoptosis, and loss of cell stiffness compared to single exposures; however, the cellular uptake of the two NPs showed no changes compared with single exposure [60].

Overall, synergistic toxicity of co-exposure to mixed NPs manifests at different levels and aspects, which arise from various mechanisms, and are closely related to the increased cellular uptake under co-exposure in many cases.

#### 3.2.2. Antagonistic Toxic Effect

Antagonistic toxic effect is manifested as the toxicity of the mixture being lower than the sum of the individual toxicities of each component exposed separately. That is, one NP reduces the toxic effects of another NP, or interferes with each other to reduce toxicity. In general, the antagonistic toxic effects of mixed NPs are mainly attributed to reduced NP uptake via blocking and competing for endocytic pathways. Inhibiting ion dissolution from NPs can further mitigate toxic damage independent of particle internalization. Liang et al. reported that ZnO NPs alone significantly induced DNA damage and inhibited the proliferation of human immortalized keratinocytes (HaCaT); however, the presence of TiO_2_ NPs greatly attenuated the above-mentioned cytotoxic effects (Figure 2c) [46]. This is because TiO_2_ NPs competitively reduced the cellular uptake of ZnO NPs and inhibited the dissolution of ZnO NPs, thereby leading to a decrease in intracellular Zn^2+^ concentration and ultimately reducing its cytotoxicity [46]. Furthermore, this combined effect was validated using the in vitro Reconstructed Human Epidermis (RHE) EpiSkin (Figure 2d). The antagonistic toxicity of mixed graphene oxide (GO) and ZnO NPs in A549 cells is because GO blocks the contact between ZnO NPs and the cell membrane, reducing their uptake by cells and thus alleviating the toxic effects of ZnO NPs on oxidative stress levels, mitochondrial membrane potential and cell membrane homeostasis, and cell viability [50]. Clear antagonistic toxicity was observed between high-density polyethylene NPs and Ag NPs in human intestinal Caco-2 and HT29MTX cells [55]. This is because the NPs mutually inhibited each other’s cellular uptake by competing for shared endocytic pathways. Consequently, the cytotoxicity of the NP mixture was significantly lower than the expected additive effect.

Rafieepour et al. reported that, as determined by a two-way ANOVA factorial design, the co-exposure to Fe_2_O_3_ NPs and SNPs (both are 20–30 nm), as well as to Fe_3_O_4_ NPs (20–30 nm) and SNPs (20–100 nm) also showed antagonistic toxicity effects in A549 cells [76,77]. However, the authors did not determine the change in NP uptake, but just discussed that the antagonistic effects may be related to the modulation of oxidative stress, as well as differences in particle surface properties and the composition of the formed protein corona [76,77].

#### 3.2.3. Dependence of Combined Toxicity on Experimental Variables

The inherent physicochemical properties of NPs, including composition, size, morphology and surface groups, as well as cell culture conditions, can alter their colloidal stability, dissolution, surface characteristics, and adsorption of NP mixtures, thereby regulating their combined toxic effects. When investigating the combined toxic effects of standard diesel exhaust particle (DEP) with ZnO NPs or CuO NPs, Zerboni et al. found that non-toxic DEP generally enhanced the toxicity of ZnO NPs but reduced that of CuO NPs [62]. Further mechanistic studies indicated that DEP promoted the agglomeration of ZnO NPs, which increased their cellular uptake, thereby amplifying Zn^2+^-mediated toxicity. However, DEP inhibited the dissolution of CuO NPs, yielding a partly antagonistic effect on CuO toxicity. The two NPs exhibit different physicochemical interactions with DEP, which alter particle agglomeration and dissolution in different ways. Li et al. reported that the overall cytotoxicity of ZnO NPs and PS NPs under co-exposure was primarily driven by ZnO NPs, while PS NPs modulated the magnitude and mode of toxicity (synergistic vs. antagonistic) [78]. They found that both spherical ZnO and triangular pyramid ZnO showed a higher toxicity when mixed with PS-COOH, while the combination of triangular pyramid ZnO and PS-NH_2_ exhibited an antagonistic effect. Further studies indicated that ZnO NPs disrupted cell membrane integrity, induced excessive ROS, and decreased mitochondrial membrane potential, culminating in cell death including apoptosis, while PS NPs showed limited impact on cell viability, mainly by eliciting moderate ROS and mitochondrial membrane potential loss (especially PS-NH_2_). Mechanistically, PS NPs alter the toxicity of ZnO NPs through Zn^2+^ adsorption and changes in colloidal stability and surface charge.

The cell type, biological endpoints, and detection methods are also important influencing factors [72,78,80]. Alabi et al. observed that the toxic effects of mixed SLNs (~142 nm) and SPIONs (~16 nm) in 12 distinct cell lines were highly variable, depending on cell type and the specific assays applied [72]. According to the calculated interaction factors from the responses of single-NP and mixed-NP exposures, the mixture exhibited strongly antagonistic effects regarding cell viability, DNA damage, and ROS generation in HEK-293 cells, yet displayed synergistic toxicity in MCF-7 cells. The striking cell-type dependency is exemplified by the different responses to the same co-exposure treatment in HEK-293, MCF-7, and MDA-MB-231 cells with regard to their mitochondrial membrane potential (Figure 2e) [72]. Unfortunately, there are no data revealing the mechanism behind it. Similarly, co-exposure to NiO NPs (16.7 nm) and Mn_3_O_4_ NPs (18.4 nm) does not result in a consistent interaction; rather, it fluctuates based on cell type, cellular state, and dose-effect level [80]. Response surface methodology was employed to analyze toxicity endpoints, and isobolograms were generated to distinguish between different interactions. Li et al. reported that, at >25 μg/mL triangular pyramid ZnO NPs, the toxic effect of mixed triangular pyramid ZnO NPs and PS-NH_2_ shifted from the antagonistic effect to an additive effect by cell counting kit-8 (CCK-8) assay, whereas it shifted from the synergistic effect to the antagonistic effect when assessed by real-time cell analysis (RTCA) [78].

In addition, the change in concentration and concentration ratio may also alter the effect of co-exposure on toxicity [78,79,80]. The combined toxicity of ZnO NPs with TiO_2_, CeO_2_, Al_2_O_3_, or Y_2_O_3_ showed synergistic or antagonistic effects on cell death depending on their concentrations. At ≤400 μg/mL, CeO_2_, TiO_2_ and Y_2_O_3_ NPs synergistically enhanced the toxicity of ZnO NPs, while Al_2_O_3_ NPs reduced the toxicity. At 800 μg/mL, these NPs reduced ZnO-induced cytotoxicity, indicating a protective (antagonistic) effect (Figure 2f) [79]. The antagonistic effects are linked to a strong reduction in Zn^2+^ release from ZnO NPs when mixed with these NPs. Similarly, at >50 μg/mL spherical ZnO NPs, combined toxicity of spherical ZnO NPs and PS-NH_2_ changed from synergistic effect to antagonistic effect according to the CCK-8 viability assay, as dispersion, surface charge and Zn adsorption changed for PS-NH_2_ [78].

In summary, the cytotoxicity of mixed NPs is a complex phenomenon that extends beyond the simple additive effects of individual components. Depending on their specific interactions, mixed NPs can drive synergistic toxicity and antagonistic toxicity, and crucially, the magnitude and specific mode of this combined toxicity are heavily dependent on experimental variables, including inherent physicochemical properties, dispersion state, absolute concentrations, mixing ratios, cell types, cell culture conditions, the specific biological endpoints detected and their detection methods. Consequently, predicting the toxicological fate of mixed NPs based solely on single-exposure profiles is unreliable. This dynamic complexity underscores the critical necessity of evaluating the biological effects of NPs in complex, multi-component exposure scenarios to accurately reflect their real-world health risks.

When interpreting combined toxicity, exposure realism should also be considered. In real environments, organisms are unlikely to encounter only pristine particles or simple binary mixtures at fixed, high concentrations. Instead, exposure is often chronic and involves low doses of multiple particle types with different aging states, surface chemistries, and associated pollutants. Furthermore, environmental transformations such as aggregation/agglomeration, dissolution, oxidation, sulfidation, photoaging and adsorption of natural organic matter or environmental colloids may substantially alter the stability, bioavailability and toxicity of NPs [81,82]. While acute high-dose designs can help to identify interaction patterns and mechanistic signals, they may exaggerate aggregation, sedimentation-driven cell association, and acute stress responses compared to chronic low-dose exposure in complex environmental matrices. Therefore, interaction patterns observed in simplified in vitro systems should be considered primarily as mechanistic hazard indicators rather than as direct quantitative predictors of environmental risk.

It should be mentioned that, from the perspective of mixture toxicology, the combined effects of mixed NPs should be formally evaluated using established reference models, such as the concentration addition model, the independent action model, combination index analysis, and isobologram analysis. However, the extension of these models to multi-NP co-exposure scenarios is limited, and only some studies have adopted it [48,52,72,74,80]. This aspect needs to be strengthened in future research.

## 4. In Vivo Responses to Mixed NPs

In vitro cellular responses only represent the initial interactions between mixed NPs and biological systems. While in vitro models can provide useful mechanistic information, many of the uptake mechanisms identified in simplified monoculture systems may not directly translate to complex tissue environments in vivo. In vivo, however, NP mixtures encounter physiological barriers, blood and lymph circulation, changing protein coronas, immune recognition, macrophage sequestration, tissue-specific retention, and clearance processes. To fully understand the risks to human health, it is necessary to transition to in vivo models that account for these complex physiological barriers, blood circulation, and immune clearance. The biological effects of mixed NPs in animals have been studied using various NP pairs under different conditions. Co-exposure results in different responses in animals compared to single exposures, including changes in biodistribution and toxicity. However, current research is limited and mainly focuses on biological phenomena, and the relevant mechanisms are not well understood because the in vivo environment is more complex than the in vitro environment. The absorption, distribution, metabolism and excretion (ADME) of NPs jointly determine their toxicological outcomes. When the body is simultaneously exposed to multiple NPs, their ADME profiles may not be a simple additive effect of the single exposures. Instead, the interactions between co-exposed NPs may alter their ADME behavior, thereby shifting toxicological outcomes. We summarize the ADME behaviors and toxicity of NPs under co-exposure (Table 3).

### 4.1. Alterations in ADME Behavior

**Figure 3:**
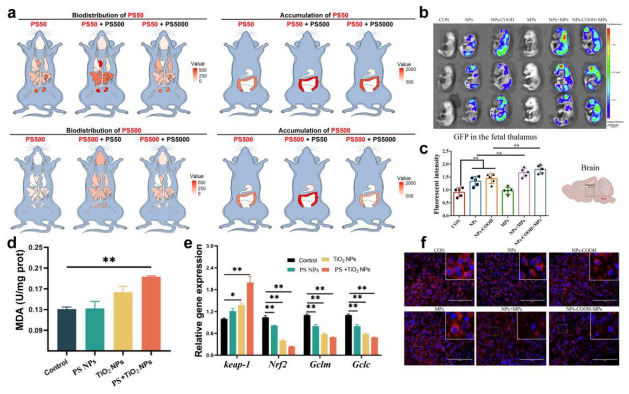
Biological effects of NP mixtures in vivo under co-exposure. (**a**) Distinct organ distribution and intestinal accumulation of 50 nm (PS50) or 500 nm (PS500) PS particles alone or in combination with other PS particles including 5000 nm PS particles (PS5000) at 24 h after a single oral administration [88] (with permission of BMC). (**b**) Fluorescence signal of PS particles in mouse fetuses after maternal oral exposure to 100 nm (NP) and/or 800 nm (MP) PS particles during gestational days 1 to 17 [75] (with permission of Elsevier). (**c**) Mean fluorescence intensity of 100 nm (NP) and/or 800 nm (MP) PS particles in the mouse fetal thalamus after maternal oral exposure during gestational days 1 to 17 (** *p* < 0.01 compared to the control group) [75] (with permission of Elsevier). (**d**) Hepatic malondialdehyde (MDA) level in mice after 28-day oral exposure to PS NPs and/or TiO_2_ NPs (** *p* < 0.01 compared to the control group) [87] (with permission of Wiley). (**e**) Relative expression of Keap-1/Nrf2/ARE-pathway-related genes in the livers of mice after 28-day oral exposure to PS and/or TiO_2_ NPs (* *p* < 0.05, ** *p* < 0.01 compared to the control group) [87] (with permission of Wiley). (**f**) Fluorescence signals of GAD2 in the mouse fetal brain after maternal oral exposure to 100 nm (NP) and/or 800 nm (MP) PS particles during gestational days 1 to 17 [75] (with permission of Elsevier).

Co-exposure can result in alterations in the absorption and distribution of NPs by disrupting physiological barriers or through competitive interactions [75,83,84,85,86,88,91,93]. When investigating the toxicity of mixed 50 nm and 500 nm PS NPs administered to mice via oral gavage, Liang et al. observed that mixed exposure led to an enhanced absorption of both PS NPs by mice [88]. They revealed that co-exposure resulted in disrupted intestinal epithelial barrier function, thereby increasing the absorption efficiency of PS NPs and resulting in their substantial accumulation in the liver, spleen, uterus, and ovaries (Figure 3a). Similarly, Zhang et al. reported that co-administration of 100 nm PS NPs and 50 nm TiO_2_ NPs via oral gavage aggravated the intestinal barrier damage in mice compared to TiO_2_ NPs exposure alone, and thus enhanced the bioaccumulation of TiO_2_ NPs in the ovaries, ultimately causing oxidative stress-mediated ovarian injury [83]. In addition, it has been reported that oral exposure to a mixture of 100 nm and 800 nm PS particles could result in the disruption of the blood-placenta barrier in pregnant mice, facilitating the translocation of NPs into fetal tissues, including the fetal brain (Figure 3b,c) [75]. Taken together, these studies confirm that disruption to barriers is a key mechanism through which the absorption efficiency of NPs is increased and their tissue translocation is accelerated under co-exposure conditions.

Competitive interactions between NPs during their tissue distribution are a key mechanism of ADME alteration. Using the ^99m^Tc labeling method, Qi et al. investigated the distribution of oxidized multi-walled carbon nanotubes (oMWCNTs, 1–10 μm in length, 10–30 nm in diameter) and nanodiamonds (NDs, 2–10 nm) in mice within 24 h after co-exposure through a single intravenous injection [91]. The results showed that although NDs did not alter the distribution of oMWCNTs, oMWCNTs affected the biodistribution of NDs in an oMWCNT dose-dependent manner. oMWCNTs decreased ND accumulation in the liver and spleen, but increased the uptake of NDs in the lungs. However, NDs had almost no effect on the distribution of oMWCNTs, revealing marked asymmetry in their mutual influence. Separately, compared with single exposure, co-exposure to Au NPs and Ag NPs of around 10 nm via intravenous injection (once a day, 5 times a week for 4 weeks) resulted in reduced accumulation of both metals in tissue, confirming competitive inhibition during clearance via the reticuloendothelial system [84]. These results suggest that exposure to multiple NPs can reduce their accumulation in primary reticuloendothelial system organs, but the magnitude of this effect varies significantly depending on the specific combination of NPs.

Besides absorption and distribution, co-exposure to multiple NPs can result in changes in the retention time and clearance rate of individual NPs in tissues. Kim et al. investigated the particle kinetics in rats after subacute inhalation exposure (6 h/day, 5 days/week for 4 weeks) to a mixture of 10 nm Ag NPs and 10 nm Au NPs (~10 μg/m^3^ each), and compared with that under single exposure (~20 μg/m^3^) [85,86]. Co-exposure resulted in changes in the lung clearance profile of Ag NPs compared to single exposure, altering the ratio of slow-to-fast clearance compartments; however, it did not affect the retention of Au NPs in most organs. Notably, the alteration of clearance also exhibited asymmetry: the clearance of Ag was accelerated while Au remained biopersistent. Unfortunately, it remained unclear whether the Ag translocated to extrapulmonary organs existed in the form of Ag NPs, Ag ions, or secondary Ag NPs formed in animals [86]. Separately, Qi et al. reported that co-exposure to oMWCNTs and NDs resulted in a delay in the excretion of both particles [91]. The reasons for this asymmetry in clearance are not well understood but it is a potentially critical factor in determining cumulative NP toxicity under chronic exposure.

### 4.2. Combined Toxicity In Vivo

In vivo studies suggest that exposure to a mixture of NPs results in three main toxicological patterns: impaired barrier function, exacerbated organ damage, and aggravated reproductive and developmental toxicity. Exposure to mixed 50 nm and 500 nm PS particles resulted in increased intestinal permeability and activated caspase-3-mediated apoptosis through the overproduction of mitochondrial ROS, ultimately leading to intestinal barrier dysfunction and enhanced NP translocation [88]. Similarly, co-exposure to PS NPs resulted in exacerbated toxicity induced by TiO_2_ NPs, including intestinal barrier damage and increased TiO_2_ accumulation in the ovaries. Ultimately, this resulted in oxidative stress-mediated ovarian injury [83].

Synergistic exacerbation of damage to the liver and kidneys via enhanced oxidative stress was observed under co-exposure to NP mixtures. Following 28 days of co-exposure to 46 nm TiO_2_ NPs and 97 nm PS NPs via oral gavage, the activities of superoxide dismutase (SOD) and glutathione (GSH) decreased, while the level of MDA (an indicator of lipid peroxidation) significantly increased and the Keap1-Nrf2-ARE signaling pathway in the liver was activated. This led to intensified hepatic apoptosis and pathology (Figure 3d,e) [87]. Similarly, following 75 days of daily oral administration of the mixture of 50 nm Al_2_O_3_ NPs and 100 nm ZnO NPs in male rats, significantly aggravated hepato-renal toxicity was observed, manifesting as worsened histopathological lesions and suppressed mitochondrial gene expression [89].

In the reproductive system, micronucleus frequency, nuclear abnormalities, and sperm abnormality rates in male mice were synergistically increased after 5 consecutive days of intraperitoneal injection of the mixed 142 nm SLNs and 112 nm SPIONs [90]. A study investigating the impact of mixed TiO_2_ and ZnO NPs on male Swiss mice revealed an overall synergistic effect on sperm parameters, though antagonism or additivity was observed at different doses for certain endpoints [92]. Furthermore, co-exposure to 100 nm and 800 nm PS particles resulted in significantly aggravated placental apoptosis, suppressed cell proliferation, reduced antioxidant capacity, and impaired γ-aminobutyric acid (GABA) synthesis in the mouse fetal thalamus. This ultimately induced anxiety-like behaviors in the offspring (Figure 3f) [75].

Crucially, these synergistic toxicological risks extend to human populations. A human experiment on cosmetics salesclerks occupationally co-exposed to ZnO (71.4 to 112 nm) and TiO_2_ (57.7 to 144.4 nm) NPs in sunscreens found significantly elevated urinary 8-hydroxy-2′-deoxyguanosine (8-OHdG) levels, with the co-exposure index significantly positively associated with both the raw and creatinine-adjusted urinary 8-OHdG concentrations, confirming the health risks of mixed NP exposure in real-world settings [96].

Co-exposure to NP mixtures can result in synergistic, antagonistic or additive effects depending on the properties of NPs and exposure conditions [93]. Co-exposure to 23.1 nm CuO NPs and 5.3 nm NiO NPs resulted in an additive inflammatory response in neutrophil percentage and ROS generation. However, 15.3 nm CB antagonized the acute pulmonary inflammatory response induced by either CuO or NiO alone. Mechanistic studies indicated that CB antagonism was due to ROS scavenging rather than competition for cellular uptake into alveolar macrophages [94]. Co-exposure to 9.3 nm CeO_2_ NPs and 38 nm DEP did not result in synergistic effect on lung inflammation but exhibited additivity in fibrotic outcomes [95].

Notably, although one study reported consistent synergism in inflammation induced by mixed NPs in both in vitro and in vivo experiments [58], most studies are either in vitro or in vivo experiments. It is rare for a study to include both cell and animal experiments. Therefore, aligning the results from cell and animal experiments is difficult. In addition, increased intracellular NP content or toxicity observed in cells may not necessarily manifest in animals due to clearance, dilution or restricted tissue penetration. Conversely, weak effects observed in monoculture systems may underestimate the risk in vivo when co-exposure disrupts biological barriers and increases systemic exposure. Furthermore, the diverse experimental conditions, including different animal models, administration methods, and dosages, make it challenging to compare the results of animal experiments with those of cell experiments. Moreover, many in vivo studies rely on bolus oral gavage, intratracheal instillation, or intravenous injection, which can reveal potential hazards, but which may not replicate continuous environmental exposure via food, drinking water, air, or occupational exposure. Therefore, the direct extrapolation of such studies to real-world risk assessment is limited unless environmentally relevant concentrations, chronic exposure schedules, transformed particles, and complex exposure matrices are considered. Future studies should establish standardized experimental protocols and conduct systematic and mechanistic investigations, as well as comprehensive dose–response investigations, to enable the predictive assessment of the mixed toxicity of NPs in the real-world.

## 5. Conclusions and Outlook

In summary, although the research on combined effects of multiple NPs is still in its early stages and lacks systematicity, we can obtain some meaningful information from existing research work. Firstly, various combined responses (antagonistic, synergistic, and unaffected effects) have been observed in both in vitro and in vivo studies. Secondly, there are many factors influencing the combined response of the mixed NPs, including but not limited to (1) the properties of individual NPs and the stability of NP mixtures, (2) the cell or animal models and their conditions, (3) the exposure conditions including NP doses and mixing ratios, exposure time, and medium, (4) the endpoints of toxicity and the measurement methods.

Nevertheless, compared with single exposure, mixed exposure is inherently more complex and difficult to understand, even for conventional pollutants (e.g., various inorganic and organic substances). The unique physicochemical complexities of NPs render co-exposure to two or more NPs exceptionally intricate and unpredictable. Currently, the related research remains limited and unsystematic. The research on the biological fates and combined toxicities of multiple NPs faces several challenges.

1. Currently, most related studies merely report experimental phenomena under simple experimental designs and without elucidating the underlying mechanisms. There are insufficient relevant research data for drawing regular or universal conclusions.
2. Critically, characterization of mixed NPs in biosystems is extremely insufficient. Mixing different NPs can result in complex interactions between them, altering their dispersion and aggregation. This not only makes it difficult to correctly analyze the combined effects observed, but also makes it almost impossible to replicate them. In addition, severe aggregation makes it difficult to attribute observed changes in uptake to genuine nanoscale interactions between NPs. Furthermore, sedimentation of large aggregates can lead to an overestimation of uptake due to gravitational settling rather than active endocytosis. Static 2D in vitro models exacerbate this issue further by failing to recapitulate dynamic flow conditions and in vivo clearance mechanisms. Currently, the inconsistent or missing characterization of NPs in biosystems under co-exposure conditions prevents meaningful comparisons.
3. The dynamic reshaping of the protein corona in mixed NP systems remains almost entirely unexplored. Key kinetic parameters, such as protein exchange rates, residence times, and competitive redistribution among co-existing NP populations have been overlooked. This critical knowledge gap currently limits our mechanistic understanding. Integrating time-resolved kinetic measurements with high-resolution proteomic profiling is necessary to determine whether the presence of one NP population alters the corona dynamics of another, and how biofluid composition influences these interactions during co-exposure.
4. Experimental designs and tested samples varied greatly across studies. Researchers used different combinations of NPs (with different compositions and properties) to measure different biological endpoints (e.g. viability, ROS, cytokine level, and apoptosis) in different biological models (e.g. different types of cells and different animal species in different states) under different experimental conditions (e.g. exposure method, time, and dose). This means that there are several variables in each study, making it difficult to attribute an observed phenomenon to a specific variable. Accordingly, it is impossible to compare experimental results from different studies.
5. The mixed NP systems studied in current laboratory settings are far removed from the real-world environmental complexity.

Looking to the future, we outline several aspects for research and development. Firstly, standardized characterization and dosimetry reporting, including delivered-to-cell dose, time-resolved aggregation profiles, medium-dependent hydrodynamic sizing, and contamination such as pyrogens and endotoxin, must be established as mandatory practice to ensure cross-study comparability and reproducibility. Standardized reporting guidelines for mixed NP exposure studies should be established in the future, including detailed characterization of the dispersion states of NP mixtures under actual exposure conditions during experimental periods. In addition, standardized dispersion protocols should be developed to improve data comparability.

Secondly, more realistic exposure conditions including chronic low-dose and appropriately matched NP ratios should be adopted to better reflect real-world environmental and occupational scenarios, rather than the acute high-dose designs that dominate current literature.

Thirdly, new techniques and methods should be adopted. High-throughput screening approaches and well-established mixture toxicology frameworks such as concentration addition, independent action, and response surface analysis should be employed in research. The applicability of these mixture toxicology models to NP systems has yet to be systematically validated. Furthermore, integrating organ-on-chip platforms and micro-physiological systems into co-exposure studies would substantially improve physiological relevance by recapitulating dynamic flow conditions, multi-organ interactions, and long-term exposure kinetics. Alongside these efforts, integrating omics-based mechanistic profiling (e.g., proteomics, transcriptomics, and metabolomics) into co-exposure studies would provide a pathway-level understanding of the molecular events underlying synergistic and antagonistic interactions.

Given that assessing the combined impacts of small chemicals is already notoriously difficult, this challenge is even greater for NPs. No matter how complicated the co-exposure to NPs is, the scientific community will certainly not be satisfied with mere case-by-case studies. Underneath the ever-changing phenomena observed in endless combinations of NP co-exposures, there are some fundamental principles and rules to be revealed. Basis on advancing data standardization and the construction of shared databases, leveraging artificial intelligence (AI) and machine learning to fill existing knowledge gaps is a crucial development direction. By training advanced AI algorithms on existing multidimensional datasets, researchers can identify hidden non-linear relationships, predict the in vivo biological fate and synergistic/antagonistic toxicities of unknown mixtures, and transition the field from observation to prediction. Combining these AI models with high-throughput screening will effectively promote the safe-by-design development and commercialization of nanotechnology. Meanwhile, future biomedical research should explore the therapeutic advantages of mixed NP systems, including their potential for synergistic drug delivery, multimodal imaging, and combination therapy, while rigorously evaluating the associated risks.

Joint efforts from materials scientists, toxicologists, computational modelers, and regulatory agencies will be essential in addressing these multifaceted challenges and advancing the field towards predictive, mechanism-based nanotoxicology.

## Figures and Tables

**Table 1:** Cellular uptake profiles of NPs under co-exposure.

Nanoparticles and Their Properties *	Cell	Experimental Conditions	Results	Ref.
SNP50: 51.0 nm (TEM), 67 nm (DLS, in medium), −27.0 mV (ZP); SNP80: 84.6 nm (TEM), 92 nm (DLS, in medium), −25.5 mV (ZP); SNP100: 104.0 nm (TEM), 118 nm (DLS, in medium), −23.5 mV (ZP); SNP150: 152.0 nm (TEM), 152 nm (DLS, in medium), −23.1 mV (ZP).	HeLa	In serum-free medium for 2 h; 2.5–40 µg/mL for each NP type.	Compared to single exposure, 100 nm SNPs markedly promoted the cellular uptake of 50 nm SNPs, whereas 50 nm SNPs inhibited the uptake of SNP100; The size-dependent interplay was also reproduced in the combinations of 50 nm SNPs + 150 nm SNPs and 80 nm SNPs + 150 nm SNPs; All SNPs were primarily internalized by HeLa cells via CME.	[45]
SNP50: 50.3 nm (TEM), 59.9 nm (DLS in medium); SNP100: 99.7 nm (TEM), 108.8 nm (DLS in medium).	HeLa; A549	In serum-free medium for 2 h; 5–53.3 µg/mL for SNP50 and SNP100, the number ratio of SNP50 to SNP100 ranged from 1 to 16.	One 100 nm SNPs triggered clathrin-mediated endocytosis and carried nearby 50 nm SNPs (up to six) into cells, enhancing the uptake of 50 nm SNPs; The number ratio of 50 nm SNPs to 100 nm SNPs inside cells was always higher than that in the medium, indicating preferential uptake of small SNPs; The size threshold for SNPs to trigger CME was one SNP of ~90 nm.	[51]
SNP40: 39.2 nm (TEM), 68.3 nm (DLS, in medium), −10.9 mV (ZP); SNP70: 69.0 nm (TEM), 82.0 nm (DLS, in medium), −22.7 mV (ZP); SNP100: 98.1 nm (TEM), 112.6 nm (DLS, in medium), −22.3 mV (ZP).	HeLa	In serum-free medium for 2 h; <60 μg/mL for all SNPs.	100 nm SNPs strongly enhanced the uptake of 40 nm SNPs (the effect weakened at higher concentrations of 40 nm SNPs. 40 nm SNPs did not enhance and might slightly inhibit the uptake of 100 nm SNPs; No significant interplay occurred at 2 μg/mL 40 nm SNPs. Mutual enhancement was observed at higher concentrations (10–20 μg/mL); In the ternary mixture, uptake of 40 nm SNPs was enhanced while uptake of 100 nm SNPs was consistently inhibited, with uptake of 70 nm SNPs being dependent on particle ratios; CME was triggered when the total volume of membrane-accumulated particles reached a ~90 nm particle equivalent threshold. achievable by single or mixed NP clusters.	[44]
PS NP (50 nm PS): 41.6 nm (TEM), 49.5 nm (DLS, in medium), −34.1 mV (ZP); PS NP (100 nm PS): 94.3 nm (TEM), 83.5 nm (DLS, in medium), −22.2 mV (ZP).	HeLa	In serum-free medium for 2 h and serum-containing medium for 2, 6, 24 h; 2.5, 5, 7.5, 10 μg/mL 50 nm PS; 10–60 μg/mL 100 nm PS; The number ratio of 50 nm PS to 100 nm PS ranged from 0.5 to 12.	Compared to single exposure, 100 nm PS enhanced uptake of 50 nm PS by up to 74.2% via triggering CME and carrying nearby 50 nm PS NPs into cells; 50 nm PS exerted complex effects on uptake of 100 nm PS (enhancement up to 71.3% or inhibition up to 38.2%) depending on concentration and mixing ratio, especially in serum-containing medium; Serum dampened short-term (2 h) particle interplay, but increased it after 24 h, leading to an increase in the uptake of 50 nm PS; CME remains dominant in both serum-free and serum-containing media; Serum protein competition modified the protein corona of 100 nm PS, causing condition-dependent changes in the uptake of 100 nm PS.	[39]
Ag NP: 34.1 nm (TEM), 34.8 nm (DLS, in medium), −13.8 mV (ZP); SNP (40 nm SNP): 42.1 nm (TEM), 69.3 nm (DLS, in medium), −32.5 mV (ZP); SNP (120 nm SNP): 119.4 nm (TEM), 117.6 nm (DLS, in medium), −26.2 mV (ZP).	HeLa	In serum-free medium for 2 h; 5 or 10 μg/mL SNPs; 2.5–15 μg/mL Ag NPs.	Co-exposure to Ag NPs and SNP led to markedly increased cellular uptake of both via CME; SNPs and Ag NPs mutually enhanced each other’s uptake: 40 nm SNPs were more effective in promoting Ag uptake at low Ag NP doses (2.5–5 μg/mL), whereas SNP120 showed stronger promotion at high Ag NP doses (≥10 μg/mL). Ag NPs strongly enhanced SNP uptake, peaking at 7.5 μg/mL; 40 nm SNPs outperformed 120 nm SNPs at high Ag NP concentrations (≥7.5 μg/mL); Ag35 and SNPs entered the same vesicles, with Ag35 and SNP40 appearing near or inside mitochondria during co-exposure.	[52]
SNP: 20.2 nm (TEM), 13.3–27.7 nm (DLS, in medium), −29.1 mV (ZP, in medium); SNP: 95.2 nm (TEM), 87.5–126.4 nm (DLS, in medium), −32.9 mV (ZP, in medium).	A549	In serum-free medium for 10–120 min; 1–30 mg/L 20 nm SNPs; 3–300 mg/L 100 nm SNPs.	Co-exposure led to concentration-dependent interactions: at low total SNP concentrations, one SNP’s uptake first increased then decreased with the other’s concentration; at high concentrations, it decreased monotonically; Under co-exposure conditions, cell-secreted proteins formed a protein corona on SNP surface, altering stability and cell surface attachment, thereby modulating uptake rates.	[40]
Carboxylated PS NP: 40 nm (TEM), 113 nm (DLS, in medium); Carboxylated PS NP: 100 nm (TEM), 171 nm (DLS, in medium).	HeLa	In serum-containing medium for 24 h; 3–100 µg/mL 40 nm PS NPs; 20–80 µg/mL 100 nm PS NPs.	40 nm PS impeded (25–100 μg/mL) or did not affect (3–12 μg/mL) the uptake of 100 nm PS, whereas 100 nm PS (20–80 μg/mL) promoted the uptake of 40 nm PS; The two PS particles partially colocalized within the cells (expected to be lysosomes based on previous studies), but they were also observed distributed independently of each other; The mechanism of this uptake modulation might have been related to differences in the biomolecular corona composition on particles of different sizes.	[41]
Ag NP: 45.4 nm (DLS, in PBS), −7.1 mV (ZP, in PBS); TAT-Ag NP: 55.6 nm (DLS, in PBS), −5.2 mV (ZP, in PBS); Au NP: 39.4 nm (DLS, in PBS), −4.9 mV (ZP, in PBS); TAT-Au NP: 38.7 nm (DLS, in PBS), −3.5 mV (ZP, in PBS); iron oxide NP: 34.2 nm (DLS, in PBS), −5.4 mV (ZP, in PBS); TAT-iron oxide NP: 36.1 nm (DLS, in PBS), −2.5 mV (ZP, in PBS); Quantum dot: 19.4 nm (DLS, in PBS), −6.3 mV (ZP, in PBS); TAT-quantum dot: 23.7 nm (DLS, in PBS), −4.2 mV (ZP, in PBS).	H1975; A549; H2122; MIA; PaCa2; CHO; HeLa; LL/2; PPC1	In serum-containing medium for 1 h; 0.27 nM Ag NPs; 0.79 nM Au NPs; 50 µg Fe/mL iron oxide NPs; 50 µg/mL quantum dots.	TAT-NPs entered cells via a receptor-dependent macropinocytosis pathway. When co-administered, they strongly induced “bystander” uptake of otherwise non-internalizing NPs (bystander NPs) of the same or different types into the same macropinosomes and endosomal compartments; The magnitude of bystander uptake increased with the concentration of either NP, while bystander NPs alone showed negligible uptake under single exposure; The two types of NP partially colocalized in intracellular vesicles under co-exposure, but could also be distributed independently within cells; Bystander uptake did not depend on physical aggregation or TAT peptide transfer between the two types of NP.	[53]
PEG-Ag NP: 10.4 nm (TEM), 40.0 nm (DLS, in PBS), −3.4 mV (ZP, in PBS); PEG-Au NP: 15.0 nm (TEM), 28.7 nm (DLS, in PBS), −5.9 mV (ZP, in PBS); TAT-Ag NP: 10.4 nm (TEM), 61.3 nm (DLS, in PBS), −3.4 mV (ZP, in PBS); TAT-Au NP: 15.0 nm (TEM), 58.5 nm (DLS, in PBS), −3.4 mV (ZP, in PBS); TAT-iron oxide NP: 3–5 nm (TEM), 69.7 nm (DLS, in PBS), −4.4 mV (ZP, in PBS).	CHO; H1975	In serum-containing medium for 1 h; 2 μL/100 μL Ag NPs and Au NPs; 50 μg Fe/mL iron oxide NPs.	One type of NP enabled strong synergistic uptake of bystander NPs, which otherwise showed little or no internalization under single exposure; The synergistic co-entry was size-dependent, peaking at a diameter of ~50 nm for both bystander Ag and Au NPs; TAT-NPs and bystander NPs were co-internalized into the same macropinosome-like vesicles regardless of B-NP size (35–102 nm); TAT-NPs significantly enhanced the apparent membrane affinity of bystander NPs during co-exposure, enabling stable membrane association not seen in single exposure.	[54]
PS NP: 30.0 nm **, 34.4–50.6 nm (DLS, in different media), −30.6 to −37.7 mV (ZP, in different media); PS NP: 100.0 nm **, 145.0–170.4 nm (DLS, in different media), −33.2 to −49.5 mV (ZP, in different media).	Caco-2; HT-29; HepG2	In serum-containing medium for 2, 6, 24 h; 100 µg/mL for both types of PS.	Under co-exposure, 30 nm PS NPs impeded the uptake of 100 nm PS NPs, and 100 nm PS NPs in turn reduced the uptake of 30 nm PS NPs; Two sizes of PS NPs competed for the same scavenger receptor-mediated pathway, mainly depending on SR-A1 (MSR1) and SR-B1 (SCARB1); Despite competition during co-exposure, 30 nm PS NPs still showed higher relative uptake efficiency than 100 nm PS NPs, preserving the size preference observed in single exposure; Co-exposure did not result in alterations in the primary internalization route, with PS NPs still entering cells primarily via scavenger receptor–mediated endocytosis rather than CME or caveolin-dependent pathway.	[47]
High-density polyethene NP: 100–200 nm (SEM), 260.6–361.82 nm (DLS, 024 h in different media), −37.9 to −30.9 mV (ZP, 024 h in PBS); Citrate-Ag NP: 20 nm, 80.2–163.4 nm (DLS, 024 h in different media), −36.7 to −28.2 mV (ZP, 024 h in PBS).	Caco-2; HT29MTX	In serum-containing medium for 2, 6, 24 h; 0.01 µg/mL polyethene NP; 40 µg/mL Ag NPs.	Polyethene NPs and Ag NPs mutually inhibited each other’s uptake, suggesting similar entry mechanisms.	[55]
SNP: 59 nm (TEM), 87 nm (DLS, in medium), −52 mV (ZP); Silica particle: 920 nm (TEM), 931 nm (DLS, in medium), −44 mV (ZP).	J774A.1	In serum-containing medium for 4, 24 h; 20 μg/mL for both types of NP.	Co-exposure (simultaneous or sequential exposure) did not result in alterations in the uptake of either 59 nm SNPs or 920 nm silica particles compared with single exposure; No colocalization was observed between 59 nm and 920 nm silica particles under co-exposure in both unstimulated and lipopolysaccharide (LPS)-stimulated macrophages; 59 nm SNPs showed strong colocalization with lysosomes during co-exposure, whereas 920 nm silica particles exhibited minimal lysosomal colocalization; LPS-induced uptake enhancement, lack of synergistic/competitive effects, and unchanged intracellular trafficking fate were all preserved under co-exposure compared with single exposure.	[56]
PMA-Au NP: 4.7 nm (TEM), 11 nm (DLS, in medium), −26 mV (ZP, in medium); PMA-FeOx NP: 13.6 nm (TEM), 28 nm (DLS, in medium), −37 mV (ZP, in medium).	J774A.1	In serum-containing medium for 124 h; 38.6 μg/mL Au NPs; 54.8 μg/mL FeOx NPs.	Co-exposure to both NPs resulted in a significantly enhanced cellular uptake by activating an alternative, compensatory uptake route utilized in single exposure and promoting crosstalk between distinct uptake mechanisms; The total cellular uptake after 24 h was increased by ~31% (Au NPs) and ~37% (FeOx NPs) under co-exposure compared with single exposure; Under co-exposure, FeOx NPs reaching lysosomes earlier than AuNPs. However, both NPs were increasingly colocalized in the same intracellular vesicles over time, while still maintaining partial separation in their intracellular distribution.	[57]
SNP: <50 nm (TEM), 1925 nm (DLS, in medium), −2.8 mV (ZP, in medium); TiO_2_ NP: <50 nm (TEM), 5066 nm (DLS, in medium), −3.3 mV (ZP, in medium).	BMDMs	In serum-containing medium for 4 h; 10 μg/cm^3^ NPs.	SiO_2_ strongly colocalized with lysosomes, whereas TiO_2_ did not. The two NPs did not colocalize.	[58]
TiO_2_ NP: ~31 nm (TEM), ~300.0 nm (DLS, in medium), ~−12.0 mV (ZP, in medium); ZnO NP: ~33 nm (TEM), 74.6–362.6 nm (DLS, in medium), −(11.2–12.8) mV (ZP, in medium).	HaCaT	In serum-containing medium for 6 and 24 h; 10, 30, 100, 200, 300 μg/mL TiO_2_ NPs; 5, 10, 20, 30, 50, 100 μg/mL ZnO NPs.	Compared with single exposure, co-exposure resulted in markedly reduced cellular uptake of Zn, including both particulate ZnO and dissolved Zn^2+^; TiO_2_ NPs induced a strong agglomeration of ZnO NPs, resulting in an increased hydrodynamic size and a reduced endocytosis of ZnO NPs; TiO_2_ NPs competed with ZnO NPs for caveolae-mediated endocytosis, limiting the uptake of non-agglomerated ZnO NPs; TiO_2_ NPs suppressed ZnO NP dissolution, leading to decreased intracellular Zn^2+^ during co-exposure.	[46]
graphene oxide (GO): 658.8 nm (DLS, in medium); ZnO NP: 50 nm (TEM), 67.8 nm (DLS, in medium).	A549	In serum-containing medium for 24 h; 1, 5, 10 mg/L GO; 10, 20, 30, 40 mg/L ZnO NPs.	GO significantly decreased the intracellular accumulation of particulate ZnO and reduced both total Zn and Zn^2+^ levels in cells; GO physically adhered to the cell membrane, blocking direct contact between ZnO NPs and the plasma membrane and thereby suppressing endocytosis and the uptake of ZnO NPs; GO did not alter the precipitation or dissolution of ZnO NPs in the culture medium, nor the primary endocytic pathway or the intracellular trafficking fate of ZnO NPs.	[50]
ZnO NP: 19 nm (TEM), 1134 nm (DLS, in medium); ZnO NP: 35 nm (TEM), 1260 nm (DLS, in medium); ZnO NP: 57 nm (TEM), 2978 nm (DLS, in medium); Cu NP: 63 nm (TEM), 265 nm (DLS, in medium).	Hep-G2	In serum-containing medium for 48 h; 6.25 μg/mL for ZnO NPs; 0.39–25.0 μg/mL for Cu NPs.	Compared with single exposure, co-exposure to ZnO NPs and Cu NPs resulted in a significantly decreased cellular content of Cu; 57 nm ZnO exerted the strongest competitive and potentiation effect; Under co-exposure, Cu NPs remained membrane-bound vesicles, while large ZnO NPs accumulated as unprocessed aggregates without membrane encapsulation; Co-exposure did not result in an altered intracellular trafficking fate of Cu NPs but disrupted the normal processing of ZnO NPs.	[59]
Citrate-Ag NP: 5 nm (TEM), stable in serum-containing DMEM media; Citrate-Pt NP: 5 nm (TEM), stable in serum-containing DMEM media.	cerebral microvascular endothelial cells (hCMEC/D3); primary astrocytes	In serum (5%)-containing medium (hCMEC/D3) and serum (10%)-containing medium (astrocyte) for 24 h; 0–50 μg/mL Ag NPs; 0–100 μg/mL Pt NPs.	During co-exposure, two types of NPs followed different or non-interfering uptake mechanisms, with no competitive uptake or mutual interference observed; Under co-exposure, the cellular uptake and trafficking of either NP in both cell types remained unchanged, including the cell-specific preference for Ag NP uptake over Pt NP uptake.	[48]
PS NP: 19.8 nm (TEM), 27.2 nm (DLS, in medium), −38.3 mV (ZP); PVP-Ag NP: 48.6 nm (TEM), 79.2 nm (DLS, in medium), −26.4 mV (ZP).	THP-1	In serum-containing medium for 2–24 h; 1, 10, 100 mg/L PS NPs; 1, 5, 10, 50 mg/L Ag NPs.	Ag NPs and PS NPs were internalized independently during co-exposure, with no alteration in uptake efficiency or quantity observed for either NP compared with single exposure; Both NPs localized mainly in the cytoplasm with no spatial overlap or aggregation between them. A small proportion of Ag NPs translocated into the nucleus, whereas PS NPs remained exclusively in the cytoplasm and did not enter the nucleus; The subcellular distribution patterns of Ag NPs and PS NPs during co-exposure were consistent with those during single exposure. There were no synergistic or competitive effects on intracellular trafficking or localization.	[49]
PVP-Ag NP: 67.1 nm (TEM), 881.5/119.5 nm (DLS, in different media), −9.3/−9.7 mV (ZP, in different media); PS NP:17.1 nm (TEM), 46.5/18.5 nm (DLS, in different media), −22.9/−9.3 mV (ZP, in different media);	Jurkat	In serum-containing or serum-free medium for 24 h; 1, 10, 100 mg/L Ag NPs; 10, 100 mg/L PS NPs.	Co-exposure did not result in a significantly altered cellular uptake of the two types of NPs compared with single exposures; Confocal imaging revealed minimal overlap in the signals of two types of NPs within cells, indicating that the two types of NPs entered and located within cells independently.	[60]
PS NP: 45.9 nm (TEM), 86.3 nm (DLS), −36.0 mV (ZP); Ag NP: 4.5 nm (TEM), 137.3 nm (DLS), −16.8 mV (ZP).	Caco-2	In serum-containing medium for 24 h; 10, 100 µg/mL PS NPs; 0.1, 0.5, 1, 5 µg/mL Ag NPs.	Compared with single exposure to Ag NPs, PS NPs were found to slightly enhance the cellular uptake of Ag (Ag NPs and Ag^+^) in a concentration-dependent manner, particularly at lower concentrations of Ag NPs; PS NPs formed stable complexes with Ag NPs in suspension. These complexes were efficiently internalized by cells and were even translocated into the nucleus.	[61]
ZnO NP: 10–40 nm (TEM), 314.4 nm (DLS, in medium), 25 mV (ZP); CuO NP: 10–50 nm (TEM), 464.7 nm (DLS, in medium), 12 mV (ZP); diesel exhaust particle (DEP): 320.8 nm (DLS, in medium), −35 mV (ZP).	A549	In serum (1%)-containing Opti-MEM for 3, 24, 48 h; 100 µg/mL DEP; 10, 15, 20, 25 µg/mL for the other NPs.	Co-exposure with DEP resulted in promoted ZnO agglomeration and thus promoted cellular uptake of ZnO NPs; Co-exposure with DEP resulted in a change in the location of CuO NPs, with them moving from the lysosomes to the phagosomes, whereas CuO NPs were predominantly found within the lysosomes under single exposure.	[62]

* Properties include TEM size, hydrodynamic size measured by DLS (in water, unless otherwise specified), zeta potential (in water, unless otherwise specified) and surface groups (if available). For DLS and ZP, “in medium” and “in PBS” indicate that the measurements were performed in cell culture medium and phosphate-buffered solution, respectively. Any values that are not integers are reported to one decimal place. ** NP sizes were provided by the manufacturer.

**Table 2:** Cytotoxicity of mixed NPs under co-exposure.

Nanoparticles and Their Properties*	Cell	Experimental Conditions	Results	Ref.
Citrate-Ag NP: 5 nm (TEM), stable in serum-containing DMEM media; Citrate-Pt NP: 5 nm (TEM), stable in serum-containing DMEM media.	cerebral microvascular endothelial cell (hCMEC/D3); primary astrocyte	In serum (5%)-containing medium (hCMEC/D3) and serum (10%)-containing medium (astrocyte) for 24 h; 0–50 μg/mL Ag NPs; 0–100 μg/mL Pt NPs.	Co-exposure led to synergistically inhibited proliferation of both cell types, especially hCMEC/D3 cells; Pt NPs significantly enhanced the toxicity of Ag NPs; Co-exposure led to far more differentially expressed proteins than either NP alone; Synergy was not due to increased NP uptake under co-exposure, but rather due to downstream cellular responses (enhanced immune-modulating responses and deregulation of extracellular matrix organization in endothelial cells).	[48]
SNP: <50 nm (TEM), 1925 nm (DLS, in medium), −2.8 mV (ZP, in medium); TiO_2_ NP: <50 nm (TEM), 5066 nm (DLS, in medium), −3.3 mV (ZP, in medium).	B6 mouse bone marrow-derived macrophages (BMDMs)	In serum-containing medium for 4 h; 10 μg/cm^3^ NPs.	The NPs, at concentrations where each NP alone did not trigger macrophage activation, synergistically upregulated IL-1β in macrophages; SNP-induced lysosomal damage and TiO_2_ NPs -induced ROS production synergistically induced macrophage cellular stress; Dispersion state of NPs may contribute to the synergistic toxic effect. The individual NPs formed big aggregates of micrometer size, but their mixture formed colloidally stable complexes (~250 nm) in the presence of divalent cations.	[58]
ZnO NP: 19 nm (TEM), 1134 nm (DLS, in medium); ZnO NP: 35 nm (TEM), 1260 nm (DLS, in medium); ZnO NP: 57 nm (TEM), 2978 nm (DLS, in medium); Cu NP: 63 nm (TEM), 265 nm (DLS, in medium).	Hep-G2	In serum-free medium for 48 h; 6.25 μg/mL ZnO NPs; 0.39–25.0 μg/mL Cu NPs.	Non-toxic ZnO NPs significantly enhanced the toxicity of Cu NPs, and the largest ZnO NPs caused the highest increase in toxicity; Co-exposure resulted in decreased intracellular Cu but increased Zn; The synergism was driven by ZnO particles, not Zn^2+^. Accumulation of large numbers of ZnO NPs in the cells altered cellular membranes and the cytotoxicity of Cu NPs was increased.	[59]
Ag NP: 34.1 nm (TEM), 34.8 nm (DLS, in medium), −13.8 mV (ZP); SNP: 42.1 nm (TEM), 69.3 nm (DLS, in medium), −32.5 mV (ZP); SNP: 119.4 nm (TEM), 117.6 nm (DLS, in medium), −26.2 mV (ZP).	HeLa	In serum-free medium for 2 h; 5 or 10 μg/mL SNPs; 2.5–15 μg/mL Ag NPs.	Co-exposure to Ag NPs and either SNP at individual non-toxic concentrations resulted in more severe cytotoxicity; The primary contributors to the combined toxicity were the internalized Ag NPs and their dissolved Ag ions that were delivered into cells by adsorbing on SNPs; Ag species triggered excessive ROS, disrupted mitochondrial function, and consequently induced apoptosis/necrosis and viability loss.	[52]
PS NP: 107.5 nm (DLS), ~−60 mV (ZP); PS-COOH NP: 129.2 nm (DLS), ~−50 mV (ZP); PS microparticle: 804.8 nm (DLS), ~−74 mV (ZP).	human choriocarcinoma HLA-G-positive cell (JEG-3)	In serum-containing medium for 24 and 48 h; 60 μg/mL PS.	Co-exposure to microparticles and non-toxic NPs resulted in more severe toxicity; Co-exposure resulted in stronger oxidative stress and injury phenotypes than single exposures, including higher ROS, higher percentage of cells at S stage, lower proliferation, higher apoptosis and lower CLDN3 (tight-junction related) level.	[75]
TiO_2_ NP: 30.8 nm (TEM), ~300 nm (DLS, in medium), ~−12 mV (ZP); ZnO NP: 33.2 nm (TEM), 74.6–362.6 nm (DLS, in medium), −(11.2–12.8) mV (ZP, in medium).	HaCaT	In serum-containing medium for 6 and 24 h; 10, 30, 100, 200, 300 μg/mL TiO_2_ NPs; 5, 10, 20, 30, 50, 100 μg/mL ZnO NPs.	Co-exposure resulted in a distinct antagonistic toxic effect in HaCaT cells; TiO_2_ NPs decreased the cellular uptake of ZnO NPs and reduced the dissolution of ZnO into Zn^2+^, leading to markedly lower intracellular total Zn and Zn^2+^ levels, and then significantly alleviating ZnO-induced reductions in cell viability, DNA damage, and skin toxicity.	[46]
graphene oxide (GO): 658.8 nm (DLS, in medium); ZnO NP: 50 nm (TEM), 67.8 nm (DLS, in medium).	A549	In serum-containing medium for 24 h; 1, 5, 10 mg/L GO; 10, 20, 30, 40 mg/L ZnO NPs.	Non-toxic GO (1–10 mg/L) reduced the bioavailability and cytotoxicity of ZnO NPs by blocking the direct contact and endocytic entry of ZnO NPs, rather than through changes in the physical state of ZnO NPs in the medium; The decreases in cell viability and increases in ROS, mitochondrial depolarization, membrane damage, and metabolic disturbances induced by ZnO NPs were all attenuated in the presence of GO.	[50]
Fe_2_O_3_ NP: 30 nm (TEM), 99.2 nm (DLS, in medium), −33.6 mV (ZP, in medium); SNP: 25 nm (TEM), 253.6 nm (DLS, in medium), −34.2 mV (ZP, in medium).	A549	In serum-containing medium for 24, 72 h; 10, 50, 100, and 250 μg/mL for each NP type.	Co-exposure resulted in an antagonistic effect; Compared with single exposures, co-exposure led to the consistent reduction in oxidative damage including lower ROS levels, weaker mitochondrial membrane depolarization and attenuated glutathione depletion.	[76]
Fe_3_O_4_ NP: 20–30 nm (TEM), 74.2 nm (>90%, DLS, in medium), −31.9 mV (ZP); SNP: 20–100 nm (TEM), 502.9 nm (>90%, DLS, in medium), −34.8 mV (ZP).	A549	In serum-containing medium for 24, 72 h; 10, 50, 100, 250 μg/mL for each NP type.	Statistically significant antagonistic interactions were detected in co-exposure; The co-exposure resulted in concentration- and time-dependent increased in cellular permeability, decreased ROS generation and glutathione content, and decreased cell viability.	[77]
High-density polyethylene NP: 100–200 nm (SEM), 260.6–361.8 nm (DLS, 0–24 h in different media), −37.9 to −30.9 mV (ZP, 0–24 h in PBS); Citrate-Ag NP: 20 nm, 80.2–163.4 nm (DLS, 0–24 h in different media), −36.7 to −28.2 mV (ZP, 0–24 h in PBS);	Caco-2; HT29MTX	In serum-containing medium for 24, 48 h; 0.01 µg/mL polyethylene NP; 15, 40 µg/mL Ag NPs.	Compared with single exposures, co-exposure to polyethylene NPs and Ag NPs resulted in less-than-additive (subtractive) toxicity, consistent with mutual uptake inhibition; Co-exposure with polyethylene NPs resulted in an attenuated toxicity induced by Ag NPs; For intracellular ROS and cell death including apoptosis, the mixed response was below the expected additive effect calculated from single exposures. Overall effects (single and mixture) were less pronounced in 3D triple-culture than in 2D culture.	[55]
PS NP: 19.8 nm (TEM), 27.2 nm (DLS, in medium), −38.3 mV (ZP); PVP-Ag NP: 48.6 nm (TEM), 79.2 nm (DLS, in medium), −26.4 mV (ZP).	THP-1	In serum-containing medium for 2–24 h; 1, 10, 100 mg/L PS NPs; 1, 5, 10, 50 mg/L Ag NPs.	The NP mixture showed additive action; The NP mixture caused stronger cell death, apoptosis, oxidative stress, mitochondrial dysfunction, and inflammatory responses than either NP alone, but did not affect anti-inflammatory cytokines; The additive toxicity arose from the sum of Ag NP-driven genotoxicity and PS NP-driven inflammatory/oxidative signaling.	[49]
PVP-Ag NP: 67.1 nm (TEM), 881.5/119.5 nm (DLS, in different media), −9.3/−9.7 mV (ZP, in different media); PS NP:17.1 nm (TEM), 46.5/18.5 nm (DLS, in different media), −22.9/−9.3 mV (ZP, in different media);	Jurkat	In serum-containing or serum-free medium for 24 h; 1, 10, 100 mg/L Ag NPs; 10, 100 mg/L PS NPs.	The mixture induced stronger oxidative stress, apoptosis and cell death than each NP alone; overall interpreted as additive toxicity; In serum-containing medium, the IC50 for Ag NP dropped from 42.9 mg/L to 4.9 and 1.37 mg/L in the presence of 10 mg/L and 100 mg/L PS NPs, respectively; In serum-containing medium, the mixtures significantly increased the intracellular ROS level, whereas single NP treatments did not; The mixture significantly reduced cell stiffness (Young’s modulus) compared with single-NP treatments at the same doses.	[60]
PMA-Au NP: 16.5 nm (TEM), 21 nm (DLS), −29.2 mV (ZP); PMA-FeOx NP: 15.8 nm (TEM), 20.1 nm (DLS), −31.4 mV (ZP).	MGC-803; A549	In serum-containing medium for 24 h; 10^−2^–10^6^ nM (Au or Fe-equivalent).	Co-exposure resulted in reduced cell viability, which was greater than that resulting from either single exposure and was within the expected range of additivity; The enhanced toxicity arose from the additive contribution of Au NP-related catalytic stress and FeOx NP-related effects.	[73]
SeO NP: 51 nm (TEM); CuO NP: 21 nm (TEM).	FLEH-104 monolayer	In serum-containing medium for 24 h; 25, 50, 100 μg/mL for each NP type.	The combined action was additive across all tested dose levels and under all mitochondrial modulation conditions.	[74]
PS NP: 45.9 nm (TEM), 86.3 nm (DLS), −36.0 mV (ZP); Ag NP: 4.5 nm (TEM), 137.3 nm (DLS), −16.8 mV (ZP).	Caco-2	In serum-containing medium for 24 h; 10, 100 µg/mL PS NPs; 0.1, 0.5, 1, 5 µg/mL Ag NPs.	Compared to single exposure, co-exposure resulted in slight alterations in some harmful cellular effects of silver, such as genotoxic damage; PS NPs could adsorb Ag NPs, forming stable complexes that entered cells and even the nucleus. However, the internalization of these complexes showed no exacerbated cytotoxic effects.	[61]
polysorbate 80-solid lipid NP (SLN): 100–300 nm (TEM), 142.0 nm (DLS); TMAH-superparamagnetic iron oxide NP (SPION): 16 nm (TEM), 112.4 nm (DLS), −38.7 mV (ZP).	NIH/3T3 MCR5; HEK-293; RWPE-1; H460; J774.1; PC-3; SK-MEL-28; MDA-MB-231; B16F10; LNCaP; MCF-7	In serum-containing medium for 24 h; 100–189 μg/mL SPIONs; 55–2200 μg/mL SLNs.	Co-exposure led to synergistic, antagonistic, and additive effects based on biotests, depending on cell type; Apoptosis triggered by ROS and disturbances in mitochondrial membrane potential were the most probable related mechanisms of action; Synergistic effects were mainly observed in tumor cell lines (PC-3, LNCaP and MCF-7); There was no significant difference between simultaneous and sequential exposure in HEK-293, MCF-7 and MDA-MB-231 cells.	[72]
PS-COOH NP: 77.8 nm (TEM), ~110 nm (DLS), ~−31 mV (ZP); PS-NH_2_ NP: 68.6 nm (TEM), ~100 nm (DLS), ~−24 mV (ZP); PEG-ZnO-S: 5.7 nm (TEM), ~1100 nm (DLS), ~−8 mV (ZP); PEG-ZnO-TP: 27.6 nm (TEM), ~500 nm (DLS), ~−13 mV (ZP).	BEAS-2B	In serum-containing medium for 24, 48, 96 h; 20, 30, 40, 50, 60 μg/mL PS NPs; 20, 30, 40, 50, 60 μg/mL ZnO NPs.	Following co-exposure, cell viability varied with ZnO morphology, the surface groups of PS, and the detection method used; The overall cytotoxic response under co-exposure was primarily driven by ZnO NPs, while PS NPs modulated the magnitude and apparent mode of toxicity (synergistic vs. antagonistic) via surface adsorption, changes in dispersion, and altered membrane interactions; Co-exposure of PS-COOH with either spherical ZnO (ZnO-S) or triangular pyramid ZnO (ZnO-TP) did not result in synergistic effects. ZnO-S and ZnO-TP were antagonistic to PS-NH_2_ according to the results of the RTCA; At >50 μg/mL ZnO-S, co-exposure to ZnO-S and PS-NH_2_ resulted in a change from synergistic to antagonistic by CCK-8, while deepened the degree of antagonism as the concentration of both increased by RTCA; At >25 μg/mL ZnO-TP, the antagonistic effect induced by the ZnO-TP and PS-NH_2_ co-exposure shifted to an additive effect by CCK-8, whereas shifted from synergistic to antagonistic effects by RTCA.	[78]
ZnO NP: 20–100 nm (TEM), 530 nm (DLS), 20.3 mV (ZP); TiO_2_ NP: 4–8 nm (TEM), 31 nm (DLS), 47.0 mV (ZP); CeO_2_ NP: 4–6 nm (TEM), 200 nm (DLS), 33.4 mV (ZP); Al_2_O_3_ NP: 12–21 nm (TEM), 312 nm (DLS), 38.0 mV (ZP); Y_2_O_3_ NP: 30–50 nm (TEM), 295 nm (DLS), 25.1 mV (ZP).	Jurkat; THP-1	Serum-containing medium for 24 h; 60 μg/mL ZnO NPs; 25, 50, 100, 200, 400, 800 μg/mL for the other NPs.	The toxic effect of co-exposure was not fixed, but concentration-dependent; At ≤400 μg/mL, CeO_2_, TiO_2_ and Y_2_O_3_ NPs synergistically enhanced the toxicity of ZnO NPs, while Al_2_O_3_ NPs reduced the toxicity. At 800 μg/mL, these NPs reduced the ZnO-induced cytotoxicity, indicating a protective (antagonistic) effect; Antagonistic effects were linked to a strong reduction in Zn^2+^ release from ZnO when mixed with these NPs; The activation of MAPK, p-38, NF-kB, and ERK depended on the combinations of ZnO NPs and the other NPs.	[79]
ZnO NP: 10–40 (TEM), 314.4 nm (DLS, in medium), 25 mV (ZP); CuO NP: 10–50 nm (TEM), 464.7 nm (DLS, in medium), 12 mV (ZP); diesel exhaust particle (DEP): 320.8 nm (DLS, in medium), −35 mV (ZP).	A549	In serum (1%)-containing Opti-MEM for 3, 24, 48 h; 100 µg/mL DEP; 10, 15, 20, 25 µg/mL for the other NPs.	Non-toxic DEP generally increased the toxicity of ZnO NPs and generally reduced that of CuO NPs; however, both mixtures markedly increased the toxicity of DEP; DEP undergo different physicochemical interactions with different metal oxide NPs, thereby altering particle agglomeration, ion release, and cellular uptake, which ultimately led to different toxicity outcomes under co-exposure; DEP promoted ZnO NPs agglomeration and cellular uptake, thereby amplifying Zn^2+^-mediated toxicity; DEP inhibited the dissolution of CuO NPs, yielding a partly antagonistic effect on CuO NP toxicity.	[62]
NiO NP: 16.7 nm (SEM), stable suspension; Mn_3_O_4_ NP: 18.4 nm (SEM), less-stable suspension (easily disaggregate).	MRC-5; THP-1; SH-SY5Y	In serum-containing medium for 24 h; 25, 50 μg/mL NiO NPs; 6.25, 12.5 μg/mL Mn_3_O_4_ NPs.	Co-exposure resulted in varied interaction depending on cell type/state and dose; In THP-1, the mixture showed super-additive features at lower-doses, more subadditive behavior at higher doses, and was closer to additivity at higher doses after macrophage differentiation; In SH-SY5Y, the mixture showed largely additive or near-additive, and the interaction strength/pattern varied with differentiation state; Serum-dependent sedimentation changed the effective co-exposure dose, which could bias mixture-effect classification.	[80]

* Properties include TEM size, hydrodynamic size measured by DLS (in water, unless otherwise specified), zeta potential (in water, unless otherwise specified) and surface groups (if available). For DLS and ZP, “in medium” and “in PBS” indicate that the measurements were performed in cell culture medium and phosphate-buffered solution, respectively. Any values that are not integers are reported to one decimal place.

**Table 3:** Distribution and toxicity of mixed NPs in vivo under co-exposure.

Nanoparticles and Their Properties *	Animal Model	Experimental Conditions	Results	Ref.
PS NP: 95.4 nm (SEM), 130.1 nm (DLS), −21.6 mV (ZP); TiO_2_ NP: 45.3 nm (SEM), 701.7 nm (DLS), −21.5 mV (ZP).	Female C57BL/6 mice (7 weeks old, 18 ± 2 g, acclimated for 1 week)	Oral gavage for 28 d; 5 μg/day PS NPs; 10 mg/kg/day TiO_2_ NPs.	Compared to exposure to TiO_2_ NPs alone, co-exposure with TiO_2_ NPs resulted in aggravated intestinal barrier damage in mice, thereby increasing the accumulation of TiO_2_ NPs in the ovary and enhancing oxidative stress; Co-exposure led to significant injury to ovarian structure and function, including an increased ovarian coefficient, aggravated granulosa cell abscission, follicle structural disarray, and decreased sex hormone levels, but there was no effect under individual exposure; Co-exposure-induced ovarian structure and function returned to normal when oxidative stress was alleviated.	[83]
Ag NP: 10.0 nm (TEM), 66.0 nm (DLS); Au NP: 12.8 nm (TEM), 33.0 nm (DLS).	Male Sprague-Dawley rats (258.12 ± 1.94 g)	Caudal vein injection, once daily, 5 days/week, for 4 weeks, followed by 4-week recovery; 10 or 100 µg/kg/day for each NP type.	Compared with single exposure, co-exposure resulted in reduced accumulation of both metals in tissues due to competitive inhibition and altered their clearance behavior; Ag clearance was enhanced under co-exposure, as indicated by decreased Ag levels in multiple tissues during recovery and a reduced Ag elimination half-life in the high-dose co-exposure group; Au showed biopersistency in mice, as indicated by little change in Au levels in multiple tissues during recovery and the appearance of Au in the brain during the recovery period in the co-exposure group; No distinctive systemic toxic effects, food consumption changes, or body-weight changes were reported during dosing or recovery periods.	[84]
Ag NP: 10.4 nm (TEM, aerosol in chamber), 10.9 nm (count median diameter); Au NP: 9.5 nm (TEM, aerosol in chamber), 10.8 nm (count median diameter).	Male Sprague-Dawley rats (273.63 ± 2.83 g)	Inhalation for 28 days (6 h/day, 5 days/week for 4 weeks) and measured on day 1 of exposure and days 1, 7, and 28 postexposure; ~20 μg/m^3^ for single exposure, each ~10 μg/m^3^ for mixed exposure.	The retention of Au NPs was unaffected by Ag NPs, but that Ag NPs were influenced by Au NPs. Compared to single exposures, the ratio of the Ag NP/Au NP retention decreased at 6 h of exposure, but increased significantly postexposure under co-exposure; Co-exposure resulted in higher clearance rates for both NPs than single exposure; For the co-exposure, the slow and fast compartments accounted for 50% each of the lung burden. However, for the single exposure, 1/3 of the lung burden was cleared by the fast rate and 2/3 of that by the slow rate.	[85]
Ag NP: 10.4 nm (TEM, aerosol in chamber), 10.9 nm (count median diameter); Au NP: 9.5 nm (TEM, aerosol in chamber), 10.8 nm (count median diameter).	Male Sprague-Dawley rats (273.63 ± 2.83 g)	Inhalation for 28 d (6 h/day, 5 days/week for 4 weeks) and measured on day 1 of exposure and days 1, 7, and 28 postexposure; 8.20 μg/m^3^ Au NPs; 8.99 μg/m^3^ Ag NPs.	Au NPs were biopersistent in multiple organs, whereas Ag NPs were rapidly cleared from most organs, persisting only in the olfactory bulb and brain until day 28 postexposure; co-exposure generally did not lead to the overturn of these patterns, though some kinetic parameters differed.	[86]
TiO_2_ NP: 46 nm (SEM), 665.5 nm (DLS), −2.5 mV (ZP); PS NP: 97 nm (SEM), 129.4 nm (DLS), −22.4 mV (ZP).	Female C57BL/6 mice (7 weeks old)	Oral gavage for 28 days; 10 mg/kg TiO_2_ NPs; ~0.05 mg/mouse PS NPs.	Compared with single exposures, co-exposure resulted in an exacerbated oxidative stress-mediated liver injury, as indicated by altered hepatic function parameters, suppressed Keap-1/Nrf2/ARE antioxidant signaling, increased hepatic Ti content, and aggravated histopathological injury.	[87]
PS NP: 50.7 nm (SEM), 54.7 nm (DLS), −38.3 mV (ZP); PS NP: 503.6 nm (SEM), 516.6 nm (DLS), −50.8 mV (ZP).	Male/female C57BL/6 J mice (8–20 g, acclimated for 1 week)	Oral gavage once (biodistribution) and daily for consecutive 28 days; 2.5–500 mg/kg for each type NP.	The absorption of both PS NPs was enhanced in mice under co-exposure and the increased amounts were due primarily to the increased permeability in the mouse intestines; Compared with single exposure, there was an enhanced toxicity under co-exposure, which was manifested by more severe intestinal barrier dysfunction caused by ROS-mediated epithelial cell apoptosis.	[88]
Al_2_O_3_ NP: 50 nm, ~45 nm (DLS); ZnO NP: 100 nm, ~75 nm (DLS).	Male Wistar albino rats (160–170 g)	Oral administration daily for consecutive 75 days; 70 mg/kg Al_2_O_3_ NPs; 100 mg/kg ZnO NPs.	Co-exposure resulted in more pronounced hepatic and renal toxicities and systemic inflammation than single exposure; Co-exposure resulted in more obvious suppression of hepatic gene expression of mitochondrial transcription factor A and peroxisome proliferator-activated receptor gamma-coactivator 1α, higher DNA fragmentation in liver, higher levels of p53, TNF-α, and IL-6 in liver and kidneys, and more pronounced histopathological changes in liver and kidneys; Compared to single exposure, the antioxidant levels in the liver and kidneys were significantly reduced, and the levels of malondialdehyde (MDA) and nitric oxide were higher under co-exposure; Co-exposure resulted in significant changes in plasma biochemical parameters.	[89]
Solid lipid NP (SLN): 100.0–300.0 nm (TEM), 142.0 nm (DLS); Superparamagnetic iron oxide NP (SPION): 16.0 nm (TEM), 112.4 nm (DLS), −38.7 mV (ZP).	Male Swiss albino mice (~37 g for micronucleus assay, ~46.5 g for sperm count and morphology assay)	Intraperitoneal injection daily for 5 consecutive days and Sampling at 24 h post last exposure and 5 weeks from the first day of exposure; 5 mg/kg SLNs; 170 μg/kg SPIONs.	Co-exposure resulted in a synergistic effect in the induction of genetic and reproductive alterations; Co-exposure resulted in the highest frequency of micronucleus and nuclear abnormalities, and the highest sperm abnormalities; Co-exposure resulted in increased activity of serum AST after five days and altered hematological parameters.	[90]
oMWCNT: 1–10 μm (length) × 10–30 nm (diameter) (TEM); Nanodiamond (ND): 2–10 nm (TEM).	Female Kunming mice (15–18 g)	Single intravenous injection and sampling at 2, 8, 16, 24 h post-injection; 500 μg/mouse NDs; 100, 500 or 800 μg/mouse oMWCNTs.	oMWCNTs affected the biodistribution of NDs, but NDs had little influence on the biodistribution and excretion pattern of oMWCNTs; oMWCNTs decreased the hepatic and splenic accumulation of NDs but increased their retention in the lungs; oMWCNTs were mainly captured by lung macrophages, while NDs accumulated in the bronchi and alveoli after co-administration; oMWCNTs delayed the excretion of NDs; NDs increased oMWCNT elimination from the blood, but reduced their urinary excretion.	[91]
TiO_2_ NP: <25 nm (TEM), 1492 nm (DLS), 2.8–5.8 mV (ZP); ZnO NP: <100 nm (TEM), 482.7 nm (DLS), 17.0–20.6 mV (ZP).	Male Swiss mice (28–32 g)	Intraperitoneal injection daily for 5 consecutive days and sampling on day 35; 9.38–75 mg/kg for each NP type.	Co-exposure resulted in more sperm abnormalities than single exposure; Co-exposure resulted in synergism for motile spermatozoa, sperm count and sperm abnormalities (except at 75 mg/kg, where an antagonistic effect was observed), as well as luteinising hormone concentration; Co-exposure resulted in antagonism for immotile spermatozoa, follicle-stimulating hormone (except at 37.5 mg/kg, where an additive effect was observed) and testosterone levels.	[92]
PS NP: 107.5 nm (DLS), ~−60 mV (ZP); PS-COOH NP: 129.2 nm (DLS), ~−50 mV (ZP); PS microparticle: 804.8 nm (DLS), ~−74 mV (ZP).	C57BL mice (8 weeks) (pregnant dams)	Intragastric gavage daily for 17 consecutive days, euthanized on day 18; 1 mg/day for each NP type.	Co-exposure resulted in aggravated fetal brain injury by disrupting the blood-placenta barrier, facilitating greater NP entry into the fetal thalamus, and inducing ROS-associated apoptosis and suppressing GABA synthesis, leading to anxiety-like behavior in offspring; The distribution of microparticles (e.g., placenta) in mice was not changed under co-exposure conditions; Compared with single exposures, co-exposure resulted in more pronounced placental apoptosis, decreased proliferation, and reduced antioxidant capacity in the fetal thalamus.	[75]
NiO NP: 16.7 nm (SEM), stable suspension; Mn_3_O_4_ NP: 18.4 nm (SEM), less-stable suspension (easily disaggregate).	Outbred white female rats (150 to 220 g)	Intraperitoneal injection (3 times/week, up to 18 injections); 0.5 mg/rat/injection.	The combined toxicity varied depending on the index assessed, showing additivity, synergism (superadditivity), or subadditivity; Co-exposure resulted in an increased Ni burden in the liver compared with single exposure to NiO NPs, and an increased urinary excretion of Mn and decreased urinary excretion of Ni compared with single exposure to M_3_O_4_ NPs and NiO NPs, respectively; In the combined group, proximal tubular injury was more pronounced and the increase in Kupffer cells was maximal; The combination caused a decrease in the red pulp to white pulp area ratio and an increase in the number of brown pigment microaggregates.	[93]
CuO NP: 23.1 nm (TEM), 363.1 nm (DLS, in PBS), 7.79 mV (ZP, in PBS); NiO NP: 5.3 nm (TEM), 953.4 nm (DLS, in PBS), −25.5 mV (ZP, in PBS); carbon black (CB): 15.3 nm (TEM), 541.5 nm (DLS, in PBS), 0.03 mV (ZP, in PBS).	Female Wistar rats (6-week-old, acclimatized for 1 week)	Single intratracheal instillation (500 μL) and analyzed at 24 h post-exposure; 16/24/48 cm^2^/rat for CuO NPs, 120/180/360 cm^2^/rat for NiO NPs and CB.	Co-exposure resulted in an additive inflammogenic effect on the percentage of neutrophils and ROS generation for CuO NPs + NiO NPs, whereas CB antagonized the acute pulmonary inflammatory response induced by either CuO NPs or NiO NPs. CB antagonism was attributed to ROS scavenging, rather than competition for cellular uptake into alveolar macrophages; Sequential vs. combined treatment showed no significant changes in neutrophils or Ni per alveolar macrophage, supporting “no uptake competition”; With NiO NPs fixed, increasing CB reduced the percentage of neutrophils, while the amount of Ni per macrophage remained unchanged, which was consistent with ROS-based antagonism.	[94]
CeO_2_ NP: 9.3 nm (SEM), 2.5 μm (DLS, in saline); diesel exhaust particle (DEP): 38 nm (SEM).	Male Sprague-Dawley rats	Single intratracheal instillation (0.3 mL) and analyzed at 1, 3, 10, 28 days post-exposure; 35 mg/kg DEP; 0.15–7 mg/kg CeO_2_ NPs.	The combination did not demonstrate synergism with regard to lung inflammation and injury, but was closer to additivity regarding fibrotic outcomes (collagen); DEP and CeO_2_ NPs co-localized in lung tissues and formed substantially larger, more continuous, dense clumps in lymph nodes than either particle alone; Day 1 ROS under co-exposure was below the summed individual effects (subadditive/antagonistic), whereas by day 28 it approached additivity; By day 28, the fibrosis/collagen induced by co-exposure was approximately the sum of the individual exposures (additive).	[95]
SNP: <50 nm (TEM), 1925 nm (DLS, in medium), −2.8 mV (ZP, in medium); TiO_2_ NP: <50 nm (TEM), 5066 nm (DLS, in medium), −3.3 mV (ZP, in medium).	Female C57BL/6N mice (6–7 weeks old)	Intratracheal instillation for 24 h; 2.5, 5, 10 mg/kg for each NP type.	At doses where SNP or TiO_2_ NPs alone induced little to no obvious inflammation, co-exposure resulted in marked pulmonary inflammation; At 5 mg/kg, the mixture induced marked inflammatory signals and Gr-1^+^ neutrophil infiltration in bronchoalveolar lavage fluid; Synergy was evident at lower doses (e.g., 2.5–5 mg/kg), but was not further enhanced at higher doses (e.g., 10 mg/kg), where SNP alone already triggered severe inflammation; While the individual NPs severely aggregated to the micrometer scale, the mixture formed stable ~250 nm complexes in the presence of divalent cations, which may have contributed to the synergistic inflammation.	[58]

* Properties include TEM size, hydrodynamic size measured by DLS (in water, unless otherwise specified), zeta potential (in water, unless otherwise specified) and surface groups (if available). For DLS and ZP, “in medium” and “in PBS” indicate that the measurements were performed in cell culture medium and phosphate buffer solution, respectively. Any values that are not integers are reported to one decimal place.

## Data Availability

No new data were created or analyzed in this study.

## Abbreviations

The following abbreviations are used in this manuscript:

ADME	Absorption, distribution, metabolism and excretion
CB	Carbon black
CME	Clathrin-mediated endocytosis
DEP	Diesel exhaust particle
DLS	Dynamic Light Scattering
GABA	γ-aminobutyric acid
GO	Graphene oxide
LPS	Lipopolysaccharide
ND	Nanodiamond
NP	Nanoparticle
oMWCNTs	Oxidized Multi-walled carbon nanotubes
PBS	Phosphate-buffered saline
PEG	Polyethylene glycol
PMA	Poly (isobutylene-alt-maleic anhydride)-graft-dodecylamine
PS	Polystyrene
PVP	Poly(vinyl)pyrrolidone
SEM	Scanning electron microscope
SLN	Solid lipid nanoparticle
SNP	Silica nanoparticle
SPION	Superparamagnetic iron oxide nanoparticle
TAT	Transactivator of transcription
TEM	Transmission electron microscopy
TMAH	Tetramethyl ammonium hydroxide
ZP	Zeta potential
